# Microbiota metabolite taurodeoxycholic acid maintains intestinal tissue residency of innate lymphoid cells via engagement with P2Y10 receptor

**DOI:** 10.1126/sciadv.adt9645

**Published:** 2025-08-22

**Authors:** Yuwei Xu, Zhen Xiong, Peikang Zhang, Runyuan Wu, Cunzhen Li, Hui Guo, Ying Du, Xiaoxiao Zhu, Dongdong Fan, Hongzhe Fan, Yong Tian, Yun Chen, Zusen Fan

**Affiliations:** ^1^ State Key Laboratory of Epigenetic Regulation and Intervention, Institute of Biophysics, Chinese Academy of Sciences, Beijing, China.; ^2^ University of Chinese Academy of Sciences, Beijing, China.; ^3^ The Affiliated Wuxi People’s Hospital of Nanjing Medical University, Wuxi People’s Hospital, Wuxi Medical Center, Wuxi; Department of Immunology, Jiangsu Key Lab of Cancer Biomarkers, Prevention and Treatment, Collaborative Innovation Center for Cancer Personalized Medicine, Nanjing Medical University, Nanjing, China.; ^4^ Faculty of Pharmaceutical Sciences, Shenzhen University of Advanced Technology, Shenzhen, China.

## Abstract

Innate lymphoid cells (ILCs) play critical roles in innate immunity, epithelial barrier protection, and tissue homeostasis. However, the maintenance machinery of intestinal tissue residency of ILCs remains elusive. Here, we show that gut microbiota is necessary for the maintenance of intestinal tissue residency of ILCs. Microbiota metabolite taurodeoxycholic acid (TDCA) binds to P2Y10 receptor on ILCs to initiate downstream Ca^2+^ and RhoA signaling pathways. TDCA-P2Y10 engagement induces *Zfp414* transcription to prime expression of CD69 and integrin αE on ILCs, leading to intestinal residency of ILCs. Moreover, decreased levels of TDCA or P2Y10 deficiency abrogates the intestinal residency of ILCs, resulting in severer intestinal inflammation. Of note, TDCA administration can enhance intestinal tissue residency of ILCs and promote protection against intestinal inflammation. Thus, TDCA might be used as a potential drug to treat patients with inflammatory bowel disease.

## Teaser

Microbiota metabolite TDCA binds P2Y10 on intestinal ILCs to maintain tissue residency through CD69 and integrin alphaE.

## INTRODUCTION

Innate lymphoid cells (ILCs) are important guardians in mucosal immunity, contributing to barrier immunity, tissue homeostasis, and immune regulation. ILCs are classified into three groups on the basis of their development and functional characteristics: group 1 (ILC1s), group 2 (ILC2s), and group 3 (ILC3s) (*1*). We also recently defined a subpopulation of regulatory ILCs called ILCreg that exist in the gut and suppress activation of ILC1s and ILC3s via interleukin-10 (IL-10) secretion, leading to protection against intestinal inflammation (*2*). Based on their secretion of specific cytokines, each group of ILCs specializes in unique innate immunity against different types of pathogens, such as intracellular pathogens, helminths, and extracellular pathogens, respectively (*3*). Tissue residency is a hallmark of intestinal ILCs. Upon seeding intestinal tissues during fetal development, ILCs maintain tissue sedentary lifestyle and replenish their populations predominantly by local expansion (*4*, *5*). Although long-term parabiosis experiments revealed that, under a steady-state condition, resident ILCs keep tissue dwelling, how the permanent resident state of ILC populations in the intestine is maintained and where ILCs detaching from intestinal tissues migrate remain elusive.

Gut microbiota plays a critical role in shaping an immune system and maintaining intestinal homeostasis. We previously showed that bacterial metabolite *N*-undecanoylglycine induces intestinal Tuft-2 cell expansion for antimicrobial immunity (*6*). Recently, accumulating evidence suggests that metabolites produced by microbiota regulate the function of ILCs (*7*). For example, tryptophan metabolites derived from microbiota promote IL-22 production of ILC3s via activating aryl hydrocarbon receptor (*8*). Likewise, short-chain fatty acids (SCFAs) produced by commensal microbiota from dietary fibers differentially affect intestinal ILCs in a subset-specific manner (*9*). They promote proliferation and IL-22 production of ILC3s, while they inhibit expansion of ILC2s through G protein–coupled receptor 41 (GPR41) (*10*). Recent studies have reported the role of another microbiota metabolite, bile acid metabolite, in regulating ILCs’ function and intestinal homeostasis. Increased levels of cholic acid activate intestinal ILC2 to produce IL-5, leading to increased infiltration of eosinophils into intestinal tissues and inducing protective type II inflammatory response against worm infection (*11*). Glycodeoxycholic acid binds to TGR5 receptor on ILC3 and activates downstream cyclic adenosine 3′,5′-monophosphate signaling pathways, resulting in activation of transcription factor (TF)GATA binding protein 3 (GATA3) and promotion of IL-22 secretion (*12*). However, whether microbiota and their metabolites sustain retention of ILCs in the intestine is unclear.

As environmental sensors, G protein–coupled receptors (GPCRs) have been recognized to maintain retention of tissue-resident lymphocytes. For example, adhesion GPCR family member-E5 (Adgre5 or CD97) ensures retention of cDC2 in the spleen through mechanosensing red blood cells (*13*). P2Y receptors belong to the GPCR superfamily and are expressed in various lymphocytes, implicating their regulatory effects on immune system in many ways, such as degranulation, phagocytic clearance, cytokine production, and lymphocytes migration (*14*–*16*). However, how P2Y receptors regulate ILCs residency remains unknown. Tissue-resident lymphocytes display unique phenotypic characteristics and rely on chemokine receptors and adhesion molecules for their retention within tissues, including CXCR6, CCR6, integrin α1, and CD69 (*5*, *17*, *18*). Notably, the lectin receptor CD69, a marker for tissue-resident cells, is widely expressed in various lymphocytes, including tissue-resident memory T (Trm), B (Brm), and natural killer (NK) cells (*17*, *19*, *20*). Here, we showed that gut microbiota is necessary for the maintenance of intestinal tissue residency of ILCs. Microbiota metabolite taurodeoxycholic acid (TDCA) is a ligand to P2Y10 receptor that is highly expressed on intestinal ILCs. TDCA-P2Y10 engagement initiates downstream Ca^2+^ and RhoA signaling pathways to promote *Zfp414* transcription, leading to elevated expression of CD69 and integrin αE (ITAE; also called CD103) on ILCs. TDCA administration can enhance intestinal tissue residency of ILCs and promote protection against intestinal inflammation.

## RESULTS

### Gut microbiota is necessary for the maintenance of ILCs intestinal residency

**Fig. 1.**
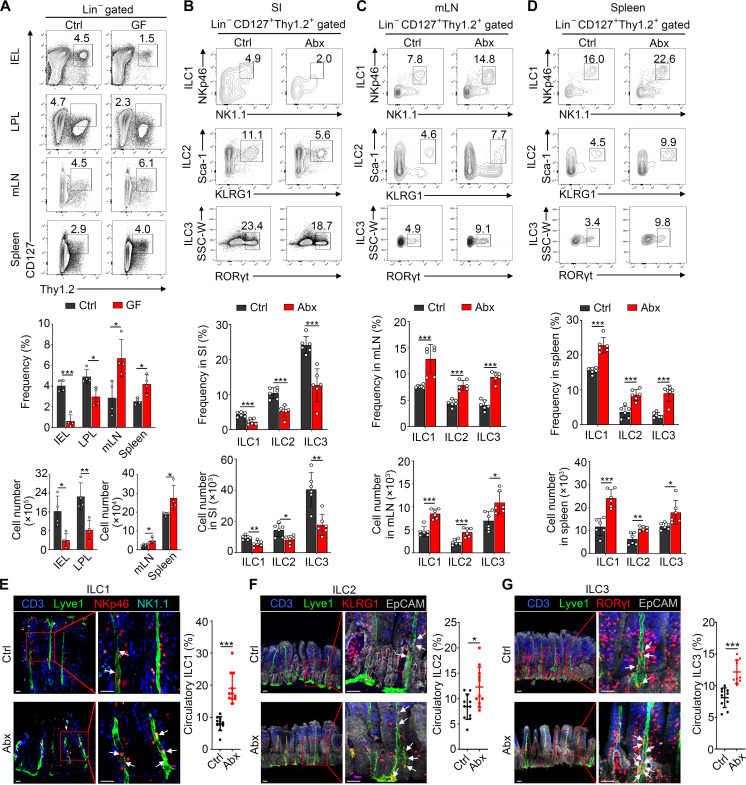
Gut microbiota is necessary for the intestinal maintenance of ILCs. (**A**) Flow cytometry analysis of total ILCs (Lin^−^Thy1.2^+^CD127^+^) from intraepithelial lymphocytes (IELs), lamina propria lymphocytes (LPLs), mesenteric lymph node (mLN), and spleen in GF and Ctrl mice. Numbers in flow cytometry plots represent percentage of ILCs in each gate. *n* = 4. (**B** to **D**) Flow cytometry analysis of ILC1s (Lin^−^CD127^+^CD45.2^+^NK1.1^+^NKp46^+^), ILC2s (Lin^−^CD127^+^Thy1.2^+^KLRG1^+^Sca-1^+^), and ILC3s (Lin^−^CD127^+^Thy1.2^+^RORγt^+^) in the SI (B), mLN (C), and spleen (D) from Ctrl and Abx-treated mice that were fed water containing a combination of antibiotics (Abx) for 2 weeks. *n* = 6. (**E** to **G**) Intestinal ILC1s (E), ILC2s (F), and ILC3s (G) located in lymphatics from Ctrl and Abx-treated mice were visualized by three-dimensional (3D) immunofluorescence staining. Lyve1 and EpCAM denote the lymphatic vessels and intestinal epithelia separately. White arrows indicate ILC subsets within lymphatic vessels. Migratory ILCs subsets were regarded as cells within lymphatic vessels. Scale bars, 70 μm. Frequencies of migratory ILC subsets in total ILCs located in a villus were calculated. Each symbol in the dot plots indicates one villus. *n* = 11. Data are representative of at least three independent experiments and are shown as the means ± SD. Statistical analysis was performed by using unpaired two-tailed Student’s *t* test (*P < 0.05; **P < 0.01; ***P < 0.001). SSC, side scatter; W, width.

To explore the effect of gut microbiota on regulating tissue residency of ILCs, we analyzed ILCs in intestinal tissues and determined their localization in germ-free (GF) mice. We found that intestinal ILCs within intestinal epithelia and lamina propria were much lower in GF mice compared with those in conventional specific pathogen–free (Ctrl) mice (Fig. 1A and fig. S1A). In addition, intestinal immunofluorescence staining showed a notable reduction in numbers of three ILC subsets in GF mice (fig. S1, B to D). Consistent with GF mice, frequencies and numbers of intestinal ILC subsets were decreased in mice treated with broad-spectrum antibiotic (Abx) (Fig. 1B and fig. S1, A and E). In addition, antibiotic treatment decreased ILC2s and ILC3s in Peyer’s patches (PPs) but had no obvious impact on ILC1s (fig. S1F). Furthermore, we found that proliferation and apoptosis rates were comparable, suggesting that ILC reduction in intestinal tissues is not caused by changes of cell proliferation and apoptosis (fig. S1G). Given that intestinal and peripheral lymphoid tissues are closely interconnected, intestinal lymphocytes are capable of trafficking between them. We then examined whether reduced intestinal ILCs migrate to lymphoid tissues such as mesenteric lymph nodes (mLNs) and spleen. We observed that percentages and numbers of ILCs from mLN and spleen were increased in GF (Fig. 1A) and Abx-treated mice (Fig. 1, C and D; and fig. S2, A and B) compared with those in Ctrl mice. It has been reported that KLRG1^+^ ILC2s represent activated ILC2s. To verify whether the reduction of intestinal ILC2s caused by Abx was due to their migration rather than activation, we sorted intestinal ILC2s for in vitro activation assays. We found that antibiotic treatment did not affect the activation of ILC2s (fig. S2, C and D). Meanwhile, we also used GATA3 as the marker for total ILC2s. Consistent with the findings on KLRG1^+^Sca-1^+^ ILC2s, antibiotic treatment decreased numbers of intestinal GATA3^+^ ILC2s, while increased in mLN and spleen (fig. S2E).

As lymphocytes egress from small intestine (SI) and enter into lymphoid tissues via lymphatic vessels (*21*), we conducted three-dimensional (3D) immunofluorescence of intestinal villi to visualize ILCs localization. Lyve1 staining was used to delineate lymphatic vessels (*22*). In a steady state, ILCs predominantly resided in lamina propria, and only a rare fraction entered into lymphatics. After Abx treatment, much more ILCs exited intestine and entered into lymphatic vessels. All of three ILC subsets displayed the same phenomena (Fig. 1, E to G). Collectively, these data indicate that microbiota environment is necessary for the maintenance of tissue residency of intestinal ILCs.

### Microbiota disruption causes migration of resident ILCs from SI to a circulation system

**Fig. 2.**
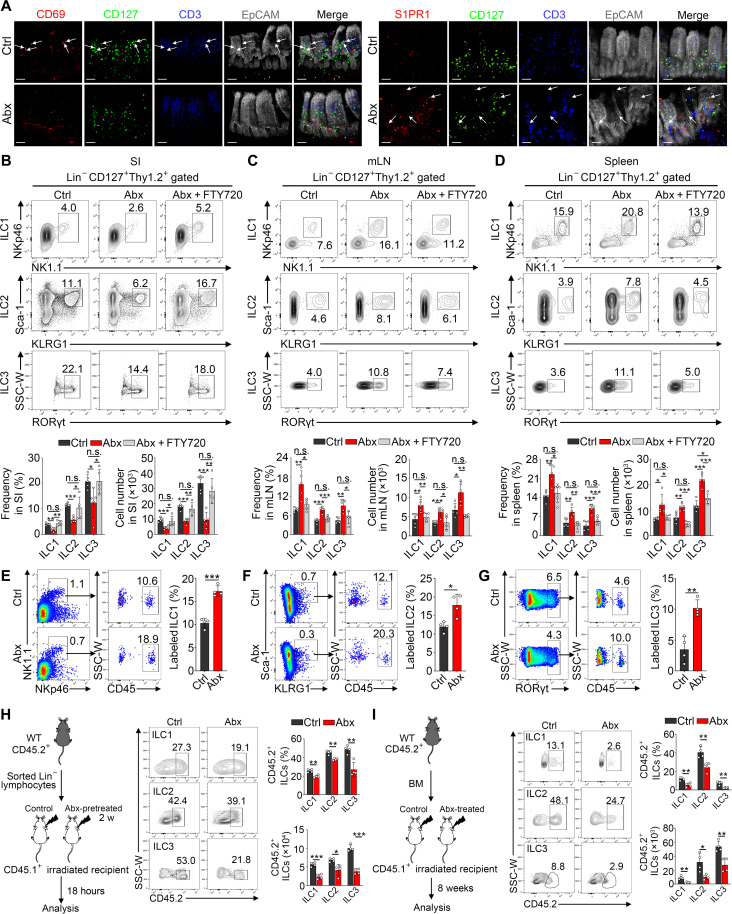
Microbiota disruption causes migration of intestinal ILCs to a circulation system. (**A**) 3D Immunofluorescence staining of CD69^+^ and S1PR1^+^ ILCs in SI from Ctrl and Abx-treated mice. White arrows denote CD69^+^ and S1PR1^+^ ILCs. Scale bars, 100 μm. (**B** to **D**) Mice were intraperitoneally injected FTY720 every other day and treated with Abx for 2 weeks. ILC1s, ILC2s, and ILC3s in SI (B), mLN (C), and spleen (D) derived from Ctrl, Abx-treated, and Abx + FTY720–treated mice were analyzed by flow cytometry. *n* = 5. (**E** to **G**) PE-labeled anti-CD45 antibody (2 μg) was intravenously injected into mice. Ten minutes later, PE-labeled intestinal ILC1s (E), ILC2s (F), and ILC3s (G) from Ctrl and Abx-treated mice were detected by flow cytometry. Frequencies of PE-labeled ILC1s, ILC2s, and ILC3s were calculated. *n* = 4. (**H**) Lin^−^ intestinal lymphocytes (1 × 10^7^) from CD45.2^+^ donor mice were sorted and intravenously injected into lethally irradiated CD45.1^+^ Ctrl or Abx-treated recipients. Eighteen hours later, donor-derived ILC subsets homing to intestine were analyzed by flow cytometry. Frequencies and numbers of intestinal CD45.2^+^ ILC subsets in indicated recipients are shown in the right panel. *n* = 4. (**I**) Bone marrow (BM) cells (5 × 10^6^) from CD45.2^+^ donor mice were intravenously injected into lethally irradiated CD45.1^+^ Ctrl or Abx-treated recipients. Eight weeks later, homing and stable localization of donor-derived ILC subsets in the intestine were analyzed by flow cytometry. Frequencies and numbers of intestinal CD45.2^+^ ILC subsets in indicated recipients are shown in the right panel. *n* = 4. Data are representative of at least three independent experiments and are shown as the means ± SD. Statistical analysis was performed by using unpaired two-tailed Student’s *t* test (*P < 0.05; **P < 0.01; ***P < 0.001; n.s., not significant). SSC, side scatter; W, width.

Previous studies have reported that S1PR1 receptor regulates migration of central memory T cells (Tcm) from peripheral nonlymphoid tissues to lymphoid tissues, and the function of S1PR1 can be antagonized by CD69 (*23*). To determine whether CD69 makes similar contribution to tissue residency of intestinal ILCs in a microbiota-dependent manner, we first examined expression of CD69 and S1PR1 on ILCs in Abx-treated versus Ctrl mice. We noticed that CD69^+^ ILCs were markedly decreased in Abx-treated mice, whereas S1PR1^+^ ILCs were increased (Fig. 2A and fig. S2, F and G). To determine that the reduction of intestinal ILCs caused by Abx treatment is due to their reentry into circulation and migration out of SI, we simultaneously treated mice with Abx and S1PR1 inhibitor FTY720 to prevent the entrance into lymphatic vessels. Flow cytometry analysis confirmed that intestinal ILC1s, ILC2s, and ILC3s were increased in Abx and FTY720 cotreated mice compared to those in mice treated with Abx alone (Fig. 2B). In contrast, three ILC subsets from mLN (Fig. 2C) and spleen (Fig. 2D) were decreased in these cotreated mice. In addition, after Abx treatment, numbers of S1PR1^+^ ILCs in the mLN and spleen increased substantially, while blockade of S1PR1 with FTY720 reduced accumulation of migratory ILCs (fig. S3A). Moreover, we sorted intestinal ILCs from tdTomato-reporter mice and performed adoptive transfer experiments. We found that, with increasing duration of antibiotic treatment, numbers of tdTomato^+^ ILCs decreased in the intestine but increased in the spleen (fig. S3, B to D). These results indicate that microbiota disruption abrogates the tissue residency of intestinal ILCs and enhances their S1PR1-dependent egression.

To directly detect the circulatory ILCs, we performed intravascular antibody staining to label cells in vascular systems. Consistent with our above observations, much more ILC1s (Fig. 2E), ILC2s (Fig. 2F), and ILC3s (Fig. 2G) from Abx-treated mice were labeled by intravascular antibody. Next, we determined whether a stable microbiota environment is necessary for homing and positioning of ILCs in SI. Intestinal Lin^−^ lymphocytes isolated from CD45.2^+^ donor mice were injected intravenously into CD45.1^+^ recipients for a short-term homing assay. In Abx recipients, ILC subsets were lower efficient in migrating to SI compared to Abx-untreated (Ctrl) recipients (Fig. 2H). Furthermore, we transferred CD45.2^+^ bone marrow (BM) cells into same CD45.1^+^ Abx recipients for a long-term homing assay to determine the importance of gut microbiota on retention of intestinal ILCs. Eight weeks later, Abx recipients showed a lower proportion and fewer numbers of intestinal ILC subsets (Fig. 2I), whereas higher percentages and greater numbers of ILC subsets were observed in mLN and spleen (fig. S3, E and F), suggesting that the intestinal environment with damaged microbiota is not conducive to homing and residency of ILCs. Together, our results reveal that disruption of gut microbiota promotes intestinal resident ILCs to exit their dwelling tissues and enter a circulation system.

### P2Y10 is highly expressed on ILCs and mediates intestinal tissue residency

**Fig. 3.**
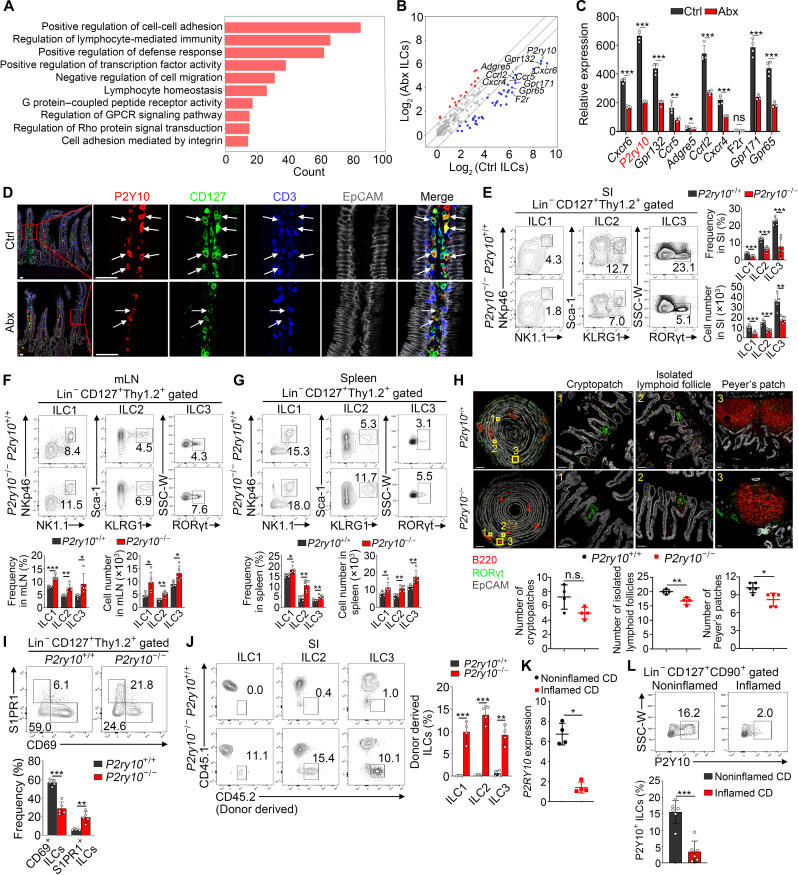
P2Y10 is highly expressed on ILCs and mediates intestinal residency of ILCs. (**A**) Transcriptome analysis of sorted intestinal ILCs (Lin^−^CD127^+^Thy1.2^+^) in Ctrl and Abx-treated mice. Highly expressed genes in Ctrl ILCs were performed GO enrichment analysis. (**B**) Expression of GPCRs was shown, and top 10 of the most specifically and highly expressed GPCRs in Ctrl ILCs were marked. (**C**) Relative mRNA levels of the candidate GPCRs in sorted Ctrl and Abx ILCs. Fold changes were normalized to *Actb*. (**D**) Immunofluorescence of P2Y10-expressing intestinal ILCs (white arrows). Scale bars, 30 μm. (**E** to **G**) Flow cytometry analysis of ILC1s, ILC2s, and ILC3s in the SI (E), mLN (F), and spleen (G) from *P2ry10*^+/+^ and *P2ry10*^−/−^ mice. *n* = 5. (**H**) Immunofluorescence of the SI from *P2ry10*^+/+^ and *P2ry10*^−/−^ mice for analysis of gut-associated lymphoid tissues. Scale bars, 2000 μm (left), 70 μm (right). *n* = 5. (**I**) Flow cytometry analysis of CD69^+^ and S1PR1^+^ ILCs derived from SI of *P2ry10*^+/+^ and *P2ry10*^−/−^ mice. *n* = 5. (**J**) *P2ry10*^+/+^ or *P2ry10*^−/−^ CD45.2^+^ donor mice underwent parabiosis surgery with CD45.1^+^ recipient mice. Flow cytometry analysis of donor-derived intestinal ILC1s, ILC2s, and ILC3s from the CD45.1^+^ parabionts. *n* = 4. (**K**) Relative mRNA levels of *P2RY10* in ILCs from noninflamed and inflamed intestinal samples of patients with CD. Fold changes were normalized to *ACTB*. *n* = 4 independent experiments. (**L**) Flow cytometry analysis of the expression of P2Y10 in intestinal ILCs from patients with CD. *n* = 6. Data are representative of at least three independent experiments and are shown as the means ± SD. Statistical analysis was performed by using unpaired two-tailed Student’s *t* test (*P < 0.05; **P < 0.01; ***P < 0.001; n.s., not significant). SSC, side scatter; W, width.

Intestinal ILCs sense different kinds of contents derived from gut microbiota, which plays a critical role in maintaining tissue homeostasis (*10*, *24*). However, it remains unclear whether gut microbiota metabolites contribute to regulate the long-term stability of intestinal ILCs in position. To test the effect of gut microbiota on regulation of ILCs transcriptome, we conducted RNA sequencing (RNA-seq) analysis of ILCs from Ctrl versus Abx-treated mice. We noticed that ILCs from Ctrl mice markedly up-regulated Trm signature genes, whereas ILCs from Abx-treated mice were more similar to Tcm (fig. S4A). We also conducted same transcriptome analysis of ILC1, ILC2, and ILC3 from single-cell RNA sequencing (scRNA-seq) data (*25*) and found that Trm signature genes were enriched in all three ILC subsets from Ctrl mice (fig. S4B). Additionally, we found that genes up-regulated in Ctrl ILCs were associated with tissue residency, mainly encoding integrins and some adhesive receptors. By contrast, genes enriched in Abx ILCs were related to cell migration and mobilization (fig. S4, C and D). Expectedly, gene ontology (GO) pathway enrichment analysis showed that pathways associated with innate immune response and cell-cell adhesion were enriched in Ctrl ILCs. GPCR activity and downstream signaling pathways displayed prominent enrichment (Fig. 3A). In recent years, numerous studies have shown that GPCRs bind to various microbial metabolites and participate in immune and metabolic regulation in the intestine. Gut microbiota typically exerts its functions by recognizing GPCRs and modulating downstream signaling (*24*, *26*, *27*). Then, we analyzed expression of GPCRs and chose top 10 GPCR genes most highly expressed in Ctrl ILCs for further quantitative polymerase chain reaction (qPCR) validation (Fig. 3B). We found that several chemokine receptors were down-regulated in ILCs from Abx-treated mice, such as Cxcr6, Ccr5, and Cxcr4, suggesting that gut microbiota modulates chemotaxis and migration of ILCs (Fig. 3C). We further noticed that another GPCR gene, *P2ry10*, was highly expressed in Ctrl ILCs, while it was reduced after Abx treatment (Fig. 3C and fig. S4, E and F). Then, we sorted various immune cells from SI and detect the expression of *P2ry10*, and we found that *P2ry10* is most abundantly expressed in ILCs, suggesting that *P2ry10* plays an important role in intestinal ILCs (fig. S4G). Moreover, mature ILC1s, ILC2s, and ILC3s exhibited higher *P2ry10* expression compared to their precursors (fig. S4H). Compared with ILCs from lymphoid tissues such as spleen and mLN, SI-derived ILCs showed higher expression of *P2ry10* (fig. S4I). Consistently, more ILCs expressing P2Y10 were observed in Ctrl mice compared with those in microbiota-deficient mice (Fig. 3D and fig. S4, J to L). These data indicate that Abx treatment suppresses resident signatures of ILCs and down-regulates *P2ry10* expression on intestinal ILCs.

To further examine the impact of P2Y10 deficiency on ILCs retention, we generated *P2ry10*-deficient mice (fig. S5A). Consistent with the phenotype of Abx-treated mice, *P2ry10*^−/−^ mice showed decreased ILC1s, ILC2s, and ILC3s in SI (Fig. 3E and fig. S5B) but increased ILC subsets in mLN (Fig. 3F and fig. S5C) and spleen (Fig. 3G and fig. S5D) compared with their *P2ry10*^+/+^ littermates. Subsequent immunofluorescence staining further confirmed the reduction of ILC1s, ILC2s, and ILC3s in the intestine of *P2ry10*^−/−^ mice (fig. S5, E to G). Given the involvement of ILC3s, particularly CCR6^+^ ILC3s in the formation of gut-associated lymphoid tissues (*28*), we used immunofluorescence staining to visualize intestinal lymphoid structures and found reduced intestinal PPs and isolated lymphoid follicles in *P2ry10*^−/−^ mice (Fig. 3H). Meanwhile, flow cytometry analysis revealed that *P2ry10* knockout reduced numbers of ILC2s and ILC3s in PPs, but no impact on ILC1s (fig. S5H). In addition, Ki67 and annexin V analysis showed that P2Y10 deficiency did not disturb proliferation and apoptosis of ILCs (fig. S5, I and J). Moreover, P2Y10 deficiency caused decreased expression of CD69 on ILCs, while expression of S1PR1 was increased (Fig. 3I and fig. S5K). Then, we conducted BM transplantation experiments to investigate whether intestinal homing and long-term residency of ILCs are dependent on P2Y10 receptor. Because *P2ry10* knockout did not affect the proportion of ILC precursors (fig. S6A), an equal number (5 × 10^6^) of BM cells from *P2ry10*^+/+^ or *P2ry10*^−/−^ were, respectively, transplanted into irradiated recipients. *P2ry10*-deficient chimeric mice showed reduced homing ILC subpopulations in SI compared with control chimeric mice (fig. S6B). Collectively, we conclude that P2Y10 receptor is required for intestinal homing and subsequent formation of long-term resident ILC populations.

In addition, we generated parabiosis mice by jointing *P2ry10*^+/+^ or *P2ry10*^−/−^ CD45.2^+^ mice with CD45.1^+^ recipient mice to confirm the impact of P2Y10 deficiency on the residency of intestinal ILCs (fig. S6C). Due to a shared circulation system, circulating T and B cells in blood and spleen could reach equilibrium between parabionts (fig. S6D). Next, we analyzed proportions of ILC1s, ILC2s, and ILC3s derived from different donor mice in recipient parabionts. We found that, compared to recipients jointed with wild-type (WT) donor mice, recipients paired with *P2ry10*^−/−^ donor mice exhibited increased proportions of CD45.2^+^ ILC subsets in SI (Fig. 3J) and blood (fig. S6E). These results indicate that loss of P2Y10 receptor disrupts residency of ILCs, leading to their entry into circulation and subsequent migration into paired parabionts via a shared circulation system. To further detect P2Y10 expression in human intestinal ILCs, we firstly analyzed RNA-seq data in patients with inflammatory bowel disease (IBD) from the Gene Expression Omnibus database (GSE57945) and observed down-regulation of *P2RY10* in patients with Crohn’s disease (CD) (fig. S7A). Subsequently, we collected ileac biopsies from endoscopically inflamed and noninflamed sites in patients with CD and then analyzed P2Y10 expression in ILCs by qPCR assay, flow cytometry, and immunofluorescence staining. We observed that most of ILCs from noninflamed intestinal samples expressed P2Y10 and residency marker CD69. However, P2Y10^+^ and CD69^+^ ILCs were decreased in inflamed intestine, while S1PR1^+^ ILCs were increased (Fig. 3, K and L; and fig. S7, B to E), suggesting that disturbed tissue residency of ILCs may contribute to the pathogenesis of intestinal inflammation. Together, P2Y10 is highly expressed on ILCs and mediates their intestinal tissue residency.

### Microbiota metabolite TDCA is a ligand to P2Y10 receptor to mediate tissue-resident signaling of intestinal ILCs

**Fig. 4.**
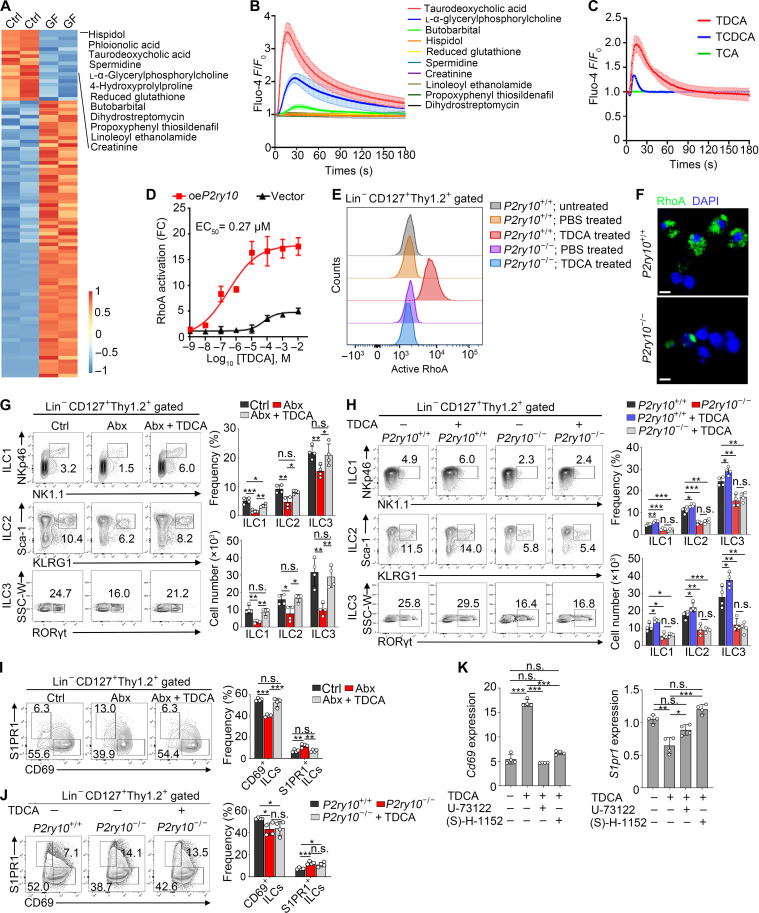
TDCA is a ligand to P2Y10 receptor. (**A**) Metabolome analysis of the intestinal contents from Ctrl and GF mice. Significantly differentiated metabolites were shown. (**B**) Response of P2Y10 to the metabolites was analyzed by detecting Ca^2+^ flux with Fluo-4 AM (2 μM). Related fluorescence intensity (*F*/*F*_0_) was calculated. *n* = 3. (**C**) Response of P2Y10 to TDCA and its metabolite precursors were analyzed as above. *n* = 3. (**D**) Response of P2Y10 to different doses of TDCA was analyzed by detecting active RhoA through luciferase reporter assay. Median effective concentration (EC_50_) was calculated. FC, fold change. (**E**) Flow cytometry analysis of RhoA activation in intestinal ILCs from *P2ry10*^+/+^ or *P2ry10*^−/−^ mice after TDCA stimulation. (**F**) Active RhoA staining in sorted intestinal ILCs from *P2ry10*^+/+^ or *P2ry10*^−/−^ mice. Scale bars, 5 μm. (**G**) Mice were orally administrated with TDCA (1 mg/20 g) daily for 2 weeks while maintained on Abx drinking. Flow cytometry analysis of intestinal ILC1s, ILC2s, and ILC3s. *n* = 4. (**H**) *P2ry10*^+/+^ and *P2ry10*^−/−^ mice kept on normal drinking were orally administrated TDCA (1 mg/20 g) daily for 2 weeks. Flow cytometry analysis of intestinal ILC1s, ILC2s, and ILC3s. *n* = 4. (**I** and **J**) Similar mice treatment as in (G) and (H) was conducted. Flow cytometry analysis of intestinal CD69^+^ and S1PR1^+^ ILCs. *n* = 4. (**K**) Intestinal ILCs were cultured with TDCA (10 μM), Ca^2+^ inhibitor (U-73122, 1 μM), or ROCK inhibitor [(S)-H-1152, 1 μM], and, then, mRNA expression of *Cd69* and *S1pr1* was analyzed. Fold changes were normalized to *Actb*. Data are representative of at least three independent experiments and are shown as the means ± SD. Statistical analysis was performed by using unpaired two-tailed Student’s *t* test (*P < 0.05; **P < 0.01; ***P < 0.001; n.s., not significant). SSC, side scatter; W, width.

We next intended to identify microbiota metabolites that engaged with P2Y10 receptor. Through untargeted metabolomics analysis on gut contents from Ctrl and GF mice, we found that disruption of microbiota caused significant alterations in the abundance of numerous metabolites. We chose metabolites specifically and highly enriched in Ctrl mice but markedly decreased in GF mice (fold change > 2) for further screening (Fig. 4A). Because P2Y10 receptor belongs to GPCRs, its activation can be assessed by detecting Ca^2+^ flux with a calcium indicator, Fluo-4 AM (*29*). Among these highly enriched metabolites, TDCA stimulation showed maximal activation of P2Y10 receptor (Fig. 4B). TDCA is a secondary bile acid that requires microbiota for its generation. We noticed that TDCA level was much lower in GF mice compared to that in Ctrl mice, while the levels of its precursors, TCDCA and TCA, were higher in GF mice (fig. S8A), suggesting that gut microbiota metabolizes TCDCA and TCA to form TDCA. In addition, TDCA was able to effectively activate P2Y10 compared to TCDCA or TCA (Fig. 4C and fig. S8B). Apart from calcium mobilization, previous studies have reported that P2Y10 receptor can also activate downstream RhoA signaling (*14*). Subsequently, G-LISA and luciferase reporter assay were used to verify the activation effect of TDCA on RhoA signaling pathway (Fig. 4D and fig. S8, C and D). Furthermore, median effective concentration for binding of TDCA to P2Y10 was 0.27 μM (Fig. 4D).

In addition, we validated the effect of TDCA on intestinal ILCs. Flow cytometry analysis showed that TDCA stimulation enhanced the level of active RhoA in *P2ry10*^+/+^ ILCs, but not in *P2ry10*^−/−^ ILCs (Fig. 4E). Consistently, immunofluorescence staining showed that ILCs from *P2ry10*^+/+^ mice exhibited a marked increase in intracellular active RhoA level after TDCA stimulation (Fig. 4F). Next, we explored whether TDCA regulates the retention of intestinal ILCs. TDCA orally administration rescued a reduction in the number of intestinal ILC subsets resulted from Abx treatment (Fig. 4G), whereas such protective effect was lacking in *P2ry10*^−/−^ mice (Fig. 4H). Of note, compared with those in the untreated group, numbers of ILC1s, ILC2s, and ILC3s in intestines of *P2ry10*^+/+^ mice slightly increased with TDCA treatment (Fig. 4H).

To further determine whether TDCA-P2Y10 interaction regulates the expression of CD69 and S1PR1, we administered TDCA to Abx-treated mice followed by flow cytometry analysis. Compared to those in mice treated with Abx alone, CD69^+^ ILCs were increased in Abx-treated mice supplemented with TDCA administration, while S1PR1^+^ ILCs were decreased (Fig. 4I and fig. S8E). By contrast, in *P2ry10*^−/−^ mice, the positive regulatory effect of TDCA on CD69 expression was abolished (Fig. 4J and fig. S8F). To determine the role of P2Y10 downstream signaling in regulating expression of resident molecules, we sorted intestinal ILCs and stimulated them with TDCA. We found that TDCA increased *Cd69* expression in sorted ILCs, while decreased *S1pr1* expression (Fig. 4K). Given that TDCA engagement with P2Y10 receptor not only stimulated Ca^2+^ flux but also activated RhoA signaling, we used a phospholipase (PLC) inhibitor (U-73122) and a ROCK inhibitor [(S)-H-1152] to separately inhibit Ca^2+^ and RhoA signaling pathways. Both U-73122 and (S)-H-1152 eliminated the positive effect of TDCA on *Cd69* expression, while relieved the inhibition on *S1pr1* expression (Fig. 4K). Collectively, microbiota metabolite TDCA mediates tissue residency of intestinal ILCs via engagement with P2Y10 receptor.

### TDCA-P2Y10 engagement initiates *Zfp414* transcription

**Fig. 5.**
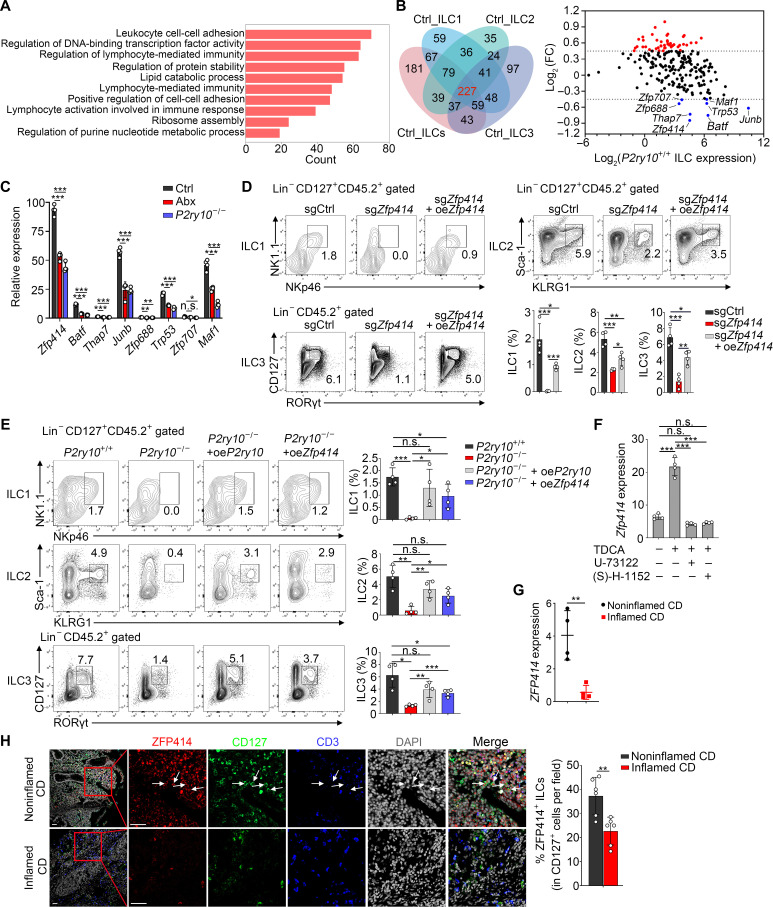
TDCA-P2Y10 engagement initiates *Zfp414* transcription. (**A**) Transcriptome analysis of sorted intestinal ILCs from *P2ry10*^+/+^ and *P2ry10*^−/−^ mice. Down-regulated genes in *P2ry10*^−/−^ ILCs were performed GO enrichment analysis. (**B**) Venn diagram of transcription factors (TFs) that were simultaneously enriched in Ctrl total ILCs and ILC subsets. The expression of TFs in intestinal ILCs from *P2ry10*^+/+^ and *P2ry10*^−/−^ mice was analyzed. FC = *P2ry10*^−/−^ ILCs/*P2ry10*^+/+^ ILCs. (**C**) Relative mRNA levels of the marked TFs above in *P2ry10*^+/+^ and *P2ry10*^−/−^ ILCs. Fold changes were normalized to *Actb*. (**D**) Construct BM chimeric mice with *Zfp414* deletion and reintroduction. Flow cytometry analysis of CD45.2^+^ intestinal ILC1s, ILC2s, and ILC3s in different BM chimeras. *n* = 4. (**E**) Construct *P2ry10*^−/−^ BM chimeras with *P2ry10* or *Zfp414* overexpression. Flow cytometry analysis of CD45.2^+^ intestinal ILC1s, ILC2s, and ILC3s in different BM chimeras. *n* = 4. (**F**) Sorted Intestinal ILCs were cultured as in Fig. 4K, and, then, the mRNA expression of *Zfp414* was analyzed. Fold changes were normalized to *Actb*. (**G**) Relative mRNA levels of *ZFP414* in ILCs from noninflamed and inflamed intestinal samples of patients with CD. Fold changes were normalized to *ACTB*. *n* = 4 independent experiments. (**H**) Immunofluorescence staining of intestinal ILCs expressing ZFP414 in noninflamed and inflamed intestinal samples of patients with CD. White arrows indicate intestinal ZFP414^+^ ILCs. Scale bars, 20 μm. Percentages of ZFP414^+^ ILCs in CD127^+^ cells per field are shown in the right panel. *n* = 6 fields. Data are representative of at least three independent experiments and are shown as the means ± SD. Statistical analysis was performed by using unpaired two-tailed Student’s *t* test (*P < 0.05; **P < 0.01; ***P < 0.001; n.s., not significant).

We intended to further define the mechanism by which TDCA-P2Y10–mediated signaling regulates long-term retention of intestinal resident ILCs. We performed RNA-seq of intestinal ILCs isolated from *P2ry10*^+/+^ and *P2ry10*^−/−^ mice. Gene set enrichment analysis (GSEA) showed that Tcm signature genes were enriched in *P2ry10*^−/−^ ILCs, while genes up-regulated in Abx-untreated ILCs were enriched in *P2ry10*^+/+^ ILCs (fig. S9A). In line with above results (Fig. 3A), several pathways such as positive regulation of cell adhesion, lymphocyte-mediated immunity, and lymphocyte activation involved in immune response were enriched in *P2ry10*^+/+^ ILCs, but not in *P2ry10*^−/−^ ILCs (Fig. 5A). Of note, the regulatory effects of TFs were attenuated in *P2ry10*^−/−^ ILCs (Fig. 5A). To further explore the TFs involved in regulation of tissue residency of ILCs, we identified 227 TFs that were simultaneously highly expressed in all ILC subsets from Abx-untreated mice. Subsequently, we analyzed expression of the 227 TFs in intestinal ILCs from *P2ry10*^+/+^ and *P2ry10*^−/−^ mice and picked out eight TFs with higher expression in *P2ry10*^+/+^ ILCs (Fig. 5B). Further qPCR validation revealed that *Zfp414* was the most highly expressed TF in Ctrl ILCs, while its expression was down-regulated after Abx treatment and *P2ry10* knockout (Fig. 5C). Subsequently, to investigate the role of ZFP414 in regulating tissue residency of intestinal ILCs, we constructed BM chimeric mice with *Zfp414* knockout using CRISPR-based mutagenesis in BM cells. *Zfp414* deficiency caused impaired homing of ILC subsets, and reintroduction of *Zfp414* rescued reduced numbers of intestinal ILC1s, ILC2s, and ILC3s (Fig. 5D and fig. S9, B and C). We further performed in vitro differentiation assay to test the effect of Zfp414 deficiency on ILC differentiation. We found that *Zfp414* depletion did not affect ILC differentiation (fig. S9D), indicating that the impaired homing of ILC subsets caused by *Zfp414* deficiency is not due to the defect of ILC differentiation. In addition, we observed that *P2ry10* reintroduction or *Zfp414* overexpression (fig. S9E) in *P2ry10*^−/−^ ILCs could increase the proportion of long-term resident ILC subsets (Fig. 5E), suggesting that ZFP414 is necessary for tissue residency of intestinal ILCs. Moreover, inhibition of P2Y10 downstream signaling pathways with U-73122 and (S)-H-1152 blocked the beneficial effect of TDCA on *Zfp414* expression (Fig. 5F). Consistent with the expression of P2Y10, ZFP414 was expressed in human ILCs derived from noninflamed intestine but down-regulated in inflamed ILCs (Fig. 5, G and H). These results indicate that TDCA-P2Y10–mediated signaling promotes the transcription of *Zfp414* for tissue residency of intestinal ILCs.

### ZFP414 activates expression of integrin αE and CD69

**Fig. 6.**
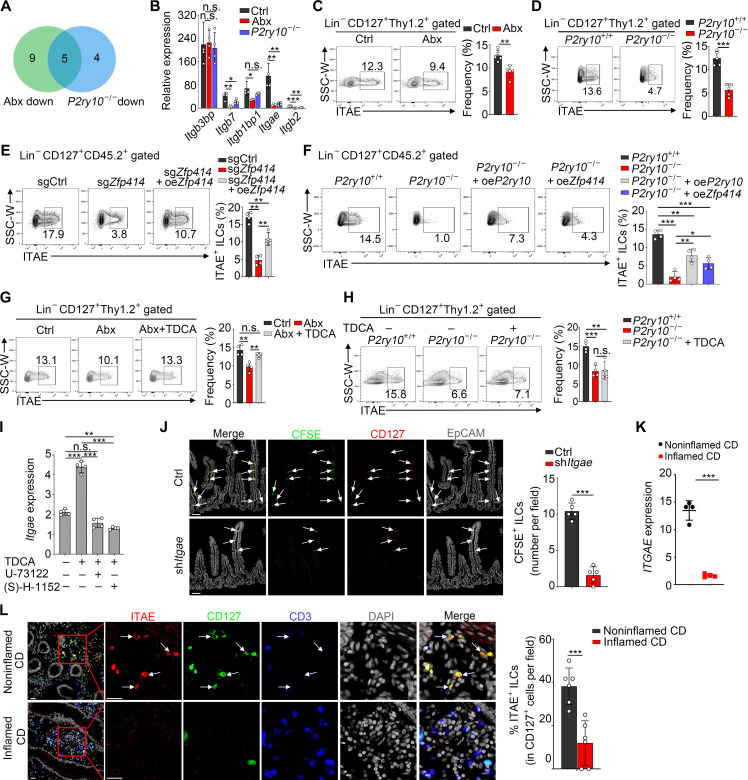
ZFP414 primes expression of integrin αE on ILCs. (**A**) Venn diagram of integrins that were simultaneously down-regulated in Abx and *P2ry10*^−/−^ ILCs. (**B**) Relative mRNA levels of integrins that were enriched in Ctrl and *P2ry10*^+/+^ ILCs. Fold changes were normalized to *Actb*. (**C** and **D**) Flow cytometry analysis of the expression of ITAE on intestinal ILCs derived from Abx-treated (C) and *p2ry10*^−/−^ mice (D). *n* = 5. (**E**) Flow cytometry analysis of the expression of ITAE on CD45.2^+^ intestinal ILCs from different BM chimeras with *Zfp414* deletion and reintroduction. *n* = 4. (**F**) Construct *P2ry10*^−/−^ BM chimeras with *P2ry10* or *Zfp414* overexpression. Flow cytometry analysis of the expression of ITAE on CD45.2^+^ intestinal ILCs from different BM chimeras. *n* = 4. (**G** and **H**) Mice were treated as in Fig. 4 (G and H). Flow cytometry analysis of the expression of ITAE on intestinal ILCs from indicated mice. *n* = 4. (**I**) Sorted intestinal ILCs were cultured as in Fig. 4K, and, then, *Itgae* expression was analyzed. Fold changes were normalized to *Actb*. (**J**) ILCs with *Itgae* silencing were labeled with CFSE (5 μM) and transferred (5 × 10^6^) into immunodeficient mice. Immunofluorescence of SI was performed. Scale bars, 70 μm. *n* = 5. (**K**) Relative mRNA levels of *ITGAE* in ILCs from noninflamed and inflamed intestinal samples of patients with CD. Fold changes were normalized to *ACTB*. *n* = 4. (**L**) Immunofluorescence of intestinal ITAE^+^ ILCs in noninflamed and inflamed intestinal samples of patients with CD. Scale bars, 20 μm. Percentages of ITAE^+^ ILCs in CD127^+^ cells per field are shown. *n* = 6 fields. Data are representative of at least three independent experiments and are shown as the means ± SD. Statistical analysis was performed by using unpaired two-tailed Student’s *t* test (*P < 0.05; **P < 0.01; ***P < 0.001; n.s., not significant). SSC, side scatter; W, width.

We next aimed to explore how TDCA-P2Y10-ZFP414 axis mediates the intestinal residency of ILCs. We observed that, besides TF activity, cell adhesion mediated by integrins showed a difference after Abx treatment (Fig. 3A). Integrins are a family of cell adhesion receptors that play a critical role in cell-cell adhesion. Previous reports have indicated that integrins play an important role in mediating tissue residency of lymphocytes (*30*, *31*). To determine the role of integrins in intestinal resident ILCs, we analyzed expression of integrins and identified five integrins that were down-regulated in Abx-treated and P2Y10-deficient ILCs (Fig. 6A). Further qPCR validation showed that *Itgae* (coding Integrin αE) and its interactor *Itgb7* displayed higher expression in untreated WT ILCs, whereas they were markedly decreased by Abx treatment and P2Y10 deficiency (Fig. 6B). Integrin αE typically forms a heterodimer by binding to integrin β7, which has been identified as a key molecule in maintaining the tissue-resident property of CD8^+^ T cells by facilitating adhesion of CD8^+^ T cells to epithelial cells (*17*). However, it is unknown whether integrin αE regulates the tissue residency of intestinal ILCs. Integrin αE exhibited lower expression in intestinal ILCs from Abx and *P2ry10*^−/−^ mice compared to those from their control littermates (Fig. 6, C and D; and fig. S9, F and G). Consistently, reduced interaction of ITAE^+^ ILCs and E-cadherin^+^ intestinal epithelia was observed in Abx and *P2ry10*^−/−^ mice (fig. S9, H and I).

To further explore whether ZFP414 regulated the expression of ITAE, we performed a chromatin immunoprecipitation (ChIP) assay with anti-ZFP414 antibody and found that ZFP414 was enriched in the *Itgae* promoter region (−400 to ~−200), suggesting that ZFP414 enriches onto the *Itgae* locus to regulate its transcription (fig. S9J). As expected, *Zfp414* deficiency resulted in decreased expression of integrin αE and CD69 in intestinal ILCs, which was restored by *Zfp414* reintroduction (Fig. 6E and fig. S10, A and B). Additionally, reintroducing *P2ry10* or overexpressing *Zfp414* in *P2ry10*^−/−^ ILCs partially rescued integrin αE and CD69 expression (Fig. 6F and fig. S10C). Consistent with the effect on CD69, TDCA administration restored expression of integrin αE in Abx ILCs, but not in *P2ry10*^−/−^ ILCs (Fig. 6, G and H; and fig. S10, D and E). Moreover, blockade of P2Y10 downstream signals eliminated the beneficial influence of TDCA on *Itgae* expression (Fig. 6I). To directly verify the function of integrin αE on ILCs positioning in the intestine, we silenced *Itgae* expression in intestinal ILCs and then transferred these ILCs into immunodeficient recipient NOD-SCID-Il2rg^−/−^ (NSG) mice to analyze their homing and resident ability. Our results showed that *Itgae* knockdown decreased ILCs homing to intestine and adhesion to epithelial cells (Fig. 6J and fig. S10F). Subsequently, we analyzed the expression of integrin αE on human ILCs with qPCR assay and immunofluorescence staining. Compared to noninflamed intestine, inflamed-intestine derived ILCs displayed much lower expression of integrin αE (Fig. 6, K and L). Together, the TDCA-P2Y10-ZFP414 axis mediates tissue residency of intestinal ILCs through integrin αE and CD69.

### Maintenance of intestinal resident ILCs contributes to protection against intestinal inflammation

**Fig. 7.**
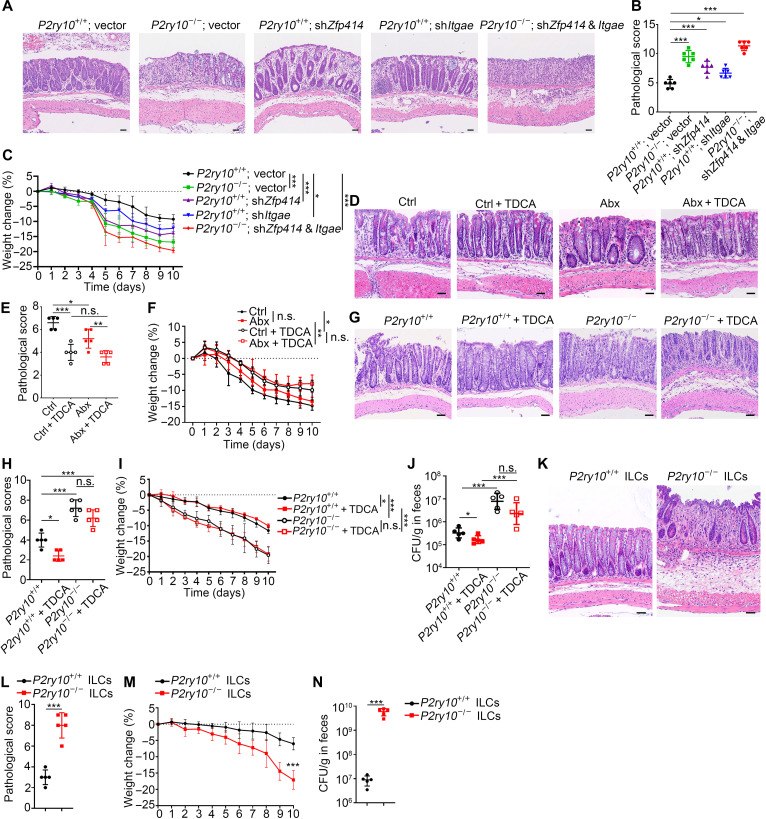
TDCA administration promotes intestinal residency of ILCs and protection against intestinal inflammation. (**A**) Mice were treated with 2% (w/v) DSS in drinking water for 5 days and followed by regular drinking water for 5 days. At day 10, colons were collected and fixed with 4% PFA for paraffin section and H&E staining. Scale bars, 50 μm. (**B** and **C**) Pathological scores (B) and weight change (C) of indicated mice. *n* = 6. (**D**) Mice were treated as in Fig. 4G, and, then, intestinal injury was induced as above with 2% (w/v) DSS. At day 10, colons were collected and fixed with 4% PFA for paraffin section and H&E staining. Scale bars, 50 μm. (**E** and **F**) Pathological scores (E) and weight change (F) of indicated mice. *n* = 5. (**G**) Mice were pretreated as in Fig. 4H and then were orally infected with *C. rodentium* (5 × 10^9^ CFU). At day 10, colons were collected for paraffin section and H&E staining. Scale bars, 50 μm. (**H** and **I**) Pathological scores (H) and body weight change (I) of indicated mice. *n* = 5. (**J**) Bacteria loads in feces were calculated. *n* = 5. (**K**) ILCs were isolated from *P2ry10*^+/+^ or *P2ry10*^−/−^ mice by flow cytometry and transferred (1 × 10^6^) into NSG mice on day −2 and 2 postinfection with *C. rodentium* (1 × 10^9^ CFU). Ten days later, colons were collected for paraffin section and H&E staining. Scale bars, 50 μm. (**L** and **M**) Pathological scores (L) and body weight change (M) of indicated mice. *n* = 5. (**N**) Bacteria loads in feces were calculated. *n* = 5. Data are representative of at least three independent experiments and are shown as the means ± SD. Statistical analysis was performed by using unpaired two-tailed Student’s *t* test (*P < 0.05; ***P < 0.001; n.s., not significant).

Intestinal resident ILCs play a key role in regulating the function of intestinal epithelial barrier, contributing to tissue homeostasis and host defense (*32*). To explore the physiological significance for maintaining stable residency of ILCs in the intestine, we generated *Zfp414*, *Itgae*-deficient mice and triple-deficient mice (sh*Zfp414*; sh*Itgae*; *P2ry10*^−/−^) through short hairpin RNA (shRNA)–based gene silencing (fig. S10G). Then, we used a dextran sulfate sodium (DSS)–induced intestinal injury model to determine whether the P2Y10-ZFP414-ITAE axis in ILCs contributes to regulation of intestinal injury and inflammation. Deficiency of *P2ry10*, *Zfp414*, or *Itgae*, respectively, showed exacerbated intestinal inflammation with DSS treatment, as evidenced by increased epithelial damage and mucosal thickness (Fig. 7A), higher pathological scores (Fig. 7B), and faster weight loss (Fig. 7C). Furthermore, simultaneous deficiencies of these three molecules caused most severe intestinal inflammation. Next, we investigated whether maintaining stable residency of ILCs by TDCA contributes to intestinal homeostasis. With DSS treatment, compared to that in the Abx-treated mice, intestinal inflammation in the control mice was slightly exacerbated, despite no notable difference in weight loss. TDCA treatment obviously alleviated intestinal inflammation in both control and Abx-treated mice (Fig. 7, D to F). In addition, to evaluate the effect of TDCA on host defense, *Citrobacter rodentium* infection model was used (*33*). *P2ry10*^−/−^ mice infected with *C. rodentium* showed more severe colitis (Fig. 7, G and H), more rapid weight loss (Fig. 7I) and more bacteria load in colons (Fig. 7J). Of note, TDCA administration protected against *C. rodentium* infection in *P2ry10*^+/+^ mice, but not in *P2ry10*^−/−^ mice (Fig. 7, G to J). To definitively validate that the increased susceptibility to intestinal inflammation in *C. rodentium* models is attributed to the decreased tissue residency of ILCs, rather than other cell types expressing P2Y10, we transferred *P2ry10*^+/+^ or *P2ry10*^−/−^ ILCs into immunodeficient NSG mice and subsequently infected mice with *C. rodentium*. We observed that *P2ry10*^−/−^ ILCs were less effective than *P2ry10*^+/+^ ILCs on protecting NSG mice and clearing *C. rodentium*, displaying more severe colitis (Fig. 7, K and L), increased weight loss (Fig. 7M), and higher bacteria load (Fig. 7N). These results indicate that maintaining tissue residency of intestinal ILCs is beneficial for intestinal homeostasis and host defense against pathogen infection.

## DISCUSSION

Tissue residency is a remarkable characteristic of ILCs. Upon reaching their destination, tissue-resident ILCs develop unique molecular characteristics influenced by local microenvironment, irrespective of their origin. However, little is known about how the tissue environment, such as the abundant microbiota in the gut, affects the stable residency of ILCs. In this study, we showed that gut microbiota is necessary for the maintenance of intestinal tissue residency of ILCs. Microbiota metabolite TDCA binds to P2Y10 receptor on ILCs to initiate downstream Ca^2+^ and RhoA signaling pathways. TDCA-P2Y10 engagement induces *Zfp414* transcription to prime expression of resident molecules CD69 and integrin αE on ILCs. These two molecules mediate the maintenance of intestinal tissue residency of ILCs by tethering ILCs to intestinal epithelial cells and restrict them out of tissue.

Microbiota and their metabolites represent an additional layer of regulation for the intestinal immune system. Microbiota-mediated signals have been defined to modulate tissue-resident lymphocytes, such as Trm and liver-resident Kupffer cells (*34*). Intestinal microbiota-derived SCFAs like acetate can directly regulate glycolytic activity of CD8^+^ T cells by GPCRs to prolong Trm survival (*35*). In addition, intestinal commensal bacteria elicit tissue-resident intestinal T helper 17 (T_H_17) cells, which are homeostatic and beneficial for barrier maintenance (*36*). Dysregulation of microbiota causes less butyrate in the liver and reduces IL-18 production in hepatocytes, leading to defective maturation of tissue-resident NK in the liver (*37*). The involvement of microbiota in regulating the function and development of intestinal ILCs were observed (*38*, *39*), but how the microbiota modulates intestinal tissue residency of ILCs has not yet been investigated. Herein, we found that all three ILC subsets were decreased in the SI of GF and Abx-treated mice, while ILC subsets were increased in lymphoid organs such as mLNs and spleen. Although total numbers of ILCs were reduced in the intestinal tracts, the proportion of ILCs in the intestinal lymphatic vessels was increased, suggesting that ILCs are no longer in a resident state and have acquired characteristics of circulation. Although conventional NK cells belong to ILC1s, we excluded them from our study and distinguished ILC1s from NK cells with CD127 expression. Now, ILC1s are known to be enriched in tissues and are generally considered to be tissue-resident cells, whereas NK cells are often regarded as circulatory cells (*40*). Furthermore, our results indicate that the expression levels of *P2ry10* in intestinal NK cells are lower than those in ILCs (fig. S3G), suggesting that P2Y10 may not play a major regulatory role in intestinal NK cells.

P2Y10 belongs to a purinergic receptor family and is classified as a GPCR due to its sevenfold transmembrane structure. Previous studies have reported that P2Y10 plays a role in the regulation of T cell migration and is expressed by other immune cells (*14*, *15*, *41*). We showed that, among intestinal immune cell populations, *P2ry10* exhibited the highest expression levels in ILCs, suggesting its important regulatory function in ILCs. In addition, we used an ILC adoptive transfer model combined with a *C. rodentium*–induced intestinal inflammation model in immunodeficient mice to exclude the role of T cells in the susceptibility to inflammation caused by *P2ry10* deficiency. We identified the gut microbiota metabolite TDCA, a secondary bile acid whose metabolism and abundance are modulated by gut microbiota (*42*), as a ligand for the P2Y10 receptor. Emerging evidence suggests that microbiota-derived secondary bile acids play a critical role in regulation of intestinal immunity (*43*, *44*). As derivatives of chlorogenic acid (LCA) and deoxycholic acid (DCA), 3-oxoLCA can directly bind to retinoid-related orphan receptor γt (RORγt) and inhibit differentiation of T_H_17 cells, while isoallo-LCA enhances the differentiation of regulatory T cells with anti-inflammatory function by promotion of Foxp3 expression (*45*). Here, we demonstrated that TDCA acts as a ligand for P2Y10 receptor. TDCA-P2Y10 engagement activates its downstream Ca^2+^ and RhoA signaling pathways to mediate tissue residency of ILCs.

Activation of GPCRs downstream Ca^2+^ and RhoA signaling pathways is involved in various biological processes. For instance, GPR55 on macrophage responds to l-α-lysophosphatidylinositol, leading to release of Ca^2+^ and activation of nuclear factor of activated T cells (*46*). The RhoA signaling pathway is also involved in the regulation of TFs transcription. RhoA activates protein kinase N to regulate the activity of myocardin-related TF A (*47*). Additionally, hyperactivate RhoA signaling is both necessary and sufficient to drive oncogenic TEAD/YAP transcription in tumor (*48*). In our study, we showed that TDCA-P2Y10–mediated Ca^2+^ and RhoA signaling pathways initiate *Zfp414* transcription to promote expression of resident molecules CD69 and integrin αE on ILCs.

In SI, CD8^+^ Trm cells inhibit SIPR1 by expressing unique molecules such as CD69 to prevent cell egression. Integrin αE binds E-cadherin on epithelial cells to tether Trm cells in the intestinal tissue. Similarly, resident T cells in the lamina propria also express CD69 and CD49a for their retention in a transforming growth factor–β–dependent manner (*49*). In this study, we showed that disruption of TDCA-P2Y10 axis down-regulates CD69 and integrin αE on intestinal ILCs, leading to their tissue egress. A recent study showed that human intestinal ILCs also exhibit resident characteristics, with low expression of *S1PR1* and *SELL* but with high expression of *CD69* (*50*). Reduced TDCA has been defined in the gut of CD (*51*). We validated that patients with CD lowly expressed P2Y10 and resident molecules, suggesting that the TDCA-P2Y10 pathway could be involved in the pathogenesis of CD. Of note, TDCA administration can enhance intestinal tissue residency of ILCs and promote protection against intestinal inflammation. Thus, TDCA could be used as a potential drug to treat patients with IBD.

## MATERIALS AND METHODS

### Antibodies and reagents

Anti-human CD3–fluorescein isothiocyanate (FITC; UCHT1, RRID: AB_314059), anti-human CD3-allophycocyanin (APC;HIT3a, RRID: AB_314047), anti-Thy1.2-APC/cyanine7 (30-H12, RRID: AB_10613280), anti-CD127-APC (SB/199, RRID: AB_1595577), anti-CD127-Brilliant Violet 711 (A7R34, RRID: AB_2564577), anti-CD127-phycoerythrin/cyanine7 (PE-cyanine7; A7R34, RRID: AB_1937266), anti-NKp46 FITC (29A1.4, RRID: AB_2149150), anti-NKp46 (29A1.4, RRID: AB_10551441), anti-CD45.1-PE-cyanine7 (A20, RRID: AB_1134170), anti-CD45.2-PE-cyanine7 (104, RRID: AB_313444), anti-CD45.2-peridinin chlorophyll protein-cyanine5.5 (PerCP-cyanine5.5; 104, RRID: AB_893352), anti–GATA3–Alexa Fluor 647 (16E10A23, RRID: AB_2563216), and anti–epithelial cell adhesion molecule (EpCAM)–Alexa Fluor 647 (G8.8, RRID: AB_1134101) were purchased from BioLegend. Anti–CD3–eFluor 450 (17A2, RRID: AB_1272193), mouse hematopoietic lineage eFluor 450 Cocktail (RRID: AB_10426799), mouse hematopoietic lineage FITC cocktail (RRID: AB_2644066), human hematopoietic lineage eFluor 450 cocktail (RRID: AB_2642588), anti–CD19–eFluor 450 (eBio1D3 (1D3), RRID: AB_2734905), anti-Thy1.2-FITC (30-H12, RRID: AB_2735033), anti-human CD90–APC–eFluor 780 (5E10, RRID: AB_2848360), anti-B220-PE (RA3-6B2, RRID: AB_465671), anti-CCR6-APC (R6H1, RRID: AB_10733388), anti-human CD127-PE (eBioRDR5, RRID: AB_10853334), anti-CD127-PE (A7R34, RRID: AB_465843), anti-CD127 (A7R34, RRID: AB_467457), anti–CD127–PerCP–eFluor 710 (SB/199, RRID: AB_2573710), anti–NK1.1–eFluor 450 (PK136, RRID: AB_2043878), anti-NK1.1-APC (PK136, RRID: AB_469479), anti-NKp46-PE-cyanine7 (29A1.4, RRID: AB_2573441), anti-NKp46-PE (29A1.4, RRID: AB_1210743), anti–Sca-1–PerCP–cyanine5.5 (D7, RRID: AB_914370), anti–Sca-1–APC (D7, RRID: AB_469486), anti-KLRG1-PE (2F1, RRID: AB_10597431), anti-KLRG1-APC (2F1, RRID: AB_469469), anti-KLRG1 (2F1, RRID: AB_469131), anti-RORγt-APC (B2D, RRID: AB_2573253), anti-RORγt (AFKJS-9, RRID: AB_1311291), anti-PLZF–Alexa Fluor 488 (Mags.21F7, RRID: AB_2574444), anti-ID2-PE (ILCID2, RRID: AB_2735055), anti–c-Kit–PE (2B8, RRID: AB_465813), anti–integrin α4β7–APC (DATK32, RRID: AB_1210577), anti–integrin α4β7–biotin (DATK32, RRID: AB_529602), anti-CD25-PE-cyanine7 (PC61.5, RRID: AB_469608), anti-Flt3-PE (A2F10, RRID: AB_465859), anti-CD45.1-FITC (A20, RRID: AB_465057), anti-CD45.1-PerCP-cyanine5.5 (A20, RRID: AB_925750), anti–CD45.1–APC–eFluor 780 (A20. RRID: AB_1582229), anti-CD45.2-PE (104, RRID: AB_465678), anti–CD45.2–APC–eFluor 780 (104, RRID: AB_1272211), anti-CD11C-FITC (N418, RRID: AB_464940), anti–CD11b–eFluor 450 (M1/70, RRID: AB_1582236), anti-F4/80-PE (BM8, RRID: AB_465923), anti-CD69-PE-cyanine7 (H1.2F3, RRID: AB_469636), anti–integrin alpha E–FITC (2E7, RRID: AB_465175), anti-Ki67-FITC (SolA15, RRID: AB_11151689), anti–IL-13–PE (eBio13A, RRID: AB_763559), anti–LYVE1–Alexa Fluor 488 (ALY7, RRID: AB_1633417), ant–LYVE1–eFluor 450 (ALY7, RRID: AB_2784723), goat anti-rabbit immunoglobulin G (IgG) (H+L)–Alexa Fluor 647 (RRID: AB_2576217), donkey anti-rabbit IgG (H+L)–Alexa Fluor 488 (RRID: AB_2535792), donkey anti-rabbit IgG (H+L)–Alexa Fluor 594 (RRID: AB_141637), goat anti-rat IgG (H+L)–Alexa Fluor 594 (RRID: AB_2535795), and goat anti-rat IgG (H+L)–Alexa Fluor 488 (RRID: AB_2535794) were purchased from eBioscience. Anti–RORγt–Brilliant Violet 421 (Q31-378, RRID: AB_2687545) and anti–IL-5–PE (TRFK-5, RRID: AB_10894193) were purchased from BD Horizon. Anti-CD69 antibody was purchased from Bioss (RRID: AB_10855614). Anti-S1PR1-PE (T4-H28, RRID: AB_10994187) was purchased from R&D Systems. Purified anti-S1PR1 antibody (RRID: AB_789722) was purchased from Novus Biologicals. Anti–E-cadherin (Ag14973, RRID: AB_10697811) was purchased from Proteintech. Purified anti-P2Y10 polyclonal antibody was purchased from Signalway Antibody and Novus. Anti-ZNF414 antibody (RRID: AB_10722362) was purchased from GeneTex. Anti–β-actin, goat anti-mouse IgG (H+L)–horseradish peroxidase (HRP) and goat anti-rabbit IgG (H+L)–HRP were purchased from Sungene Biotech. Recombinant murine IL-3, recombinant murine IL-6, recombinant murine IL-7, recombinant murine IL-2, recombinant murine IL-25, recombinant murine IL-33, and recombinant murine stem cell factor (SCF) were purchased from PeproTech. Fluo-4 AM was purchased from Yeasen. Type II collagenase and type III collagenase were purchased from Worthington. 4′,6-Diamidino-2-phenylindole (DAPI), Percoll, and paraformaldehyde (PFA) were purchased from Sigma-Aldrich. TRIzol, Lipofectamine 3000 transfection reagent, and the FoxP3 Transcription Factor Staining Kit were purchased from Thermo Fisher Scientific. Hispidol, spermidine, and dihydrostreptomycin were purchased from Psaitong. l-α-Glycerophosphorylcholine was purchased from Sigma-Aldrich. Butobarbital was purchased from Toronto Research Chemicals. Propoxyphenyl thiosildenafil and creatinine were purchased from Aladdin. Linoleoyl ethanolamide was purchased from TargetMol. Taurochenodeoxycholic acid, glutathione reduced, taurocholic acid, and fibronectin (FN) were purchased from Solarbio. TDCA was purchased from APExBIO. (S)-H-1152 was purchased from Cayman. U-73122 was purchased from MedChemExpress. DSS was purchased from MP Biomedicals. An RhoA G-LISA activation kit was purchased from Cytoskeleton. ONE-Glo Luciferase Assay System was purchased from Promega. The Annexin V Apoptosis Detection Kit was purchased from eBioscience.

### Mice

GF C57BL/6 and NSG mice were purchased from GemPharmatech Co. Ltd. (Nanjing, China). *P2ry10*^−/−^ mice were generated on the basis of the methods described before (*6*). In brief, mixtures of Cas9 mRNA (100 ng/ml) and single guide RNA (sgRNA; 50 ng/ml) (table S1) were microinjected into the cytoplasm of C57BL/6 fertilized eggs, followed by transferring to the uterus of pseudo-pregnant ICR females, from which viable founder mice were obtained. The following primers were used for deleted locus detection: 5′-TGTGCTGTGGGTCCTGTGTCCAAAGT-3′ and 5′-TATTGAAACTTTTGTGTCATTTTGTC-3′.

For generation of mice with *Itgae* and *Zfp414* silencing, sh*Itgae* (5′-GGTCATGAACTGCAAGATT-3′) and sh*Zfp414* (5′-GCTACTTCAAGTGTGAGAA-3′) were designed on online with RNAi designer (Merck or Thermo Fisher Scientific) and cloned into pLVshRNA-Puro lentivirus vector. Human embryonic kidney (HEK) 293T cell line (American Type Culture Collection, CRL-3216) was used for lentivirus packaging. When cells were 80 to 90% confluent, 8 μg of pLVshRNA-Puro plasmid, 6 μg of packaging plasmid psPAX2, and 2 μg of envelope plasmid pMD2.G were mixed and transfected into HEK293T cells by using Lipofectamine 3000 reagent. Cell supernatants were collected on the first, second, and third days after transfection and filtered through a 0.45-μm filter. Then, supernatants containing virus particles were ultracentrifuged at 25,000 rpm for 2 hours at 4°C for viral concentration. The virus pellets were resuspended with phosphate-buffered saline (PBS) and injected intravenously into *P2ry10*^+/+^ or *P2ry10*^−/−^ mice. Virus injections were performed every other day and continued for 2 weeks.

Cas9-KI, PLZF^GFPcre^, and *Id2*^+/GFP^ mice were purchased from the Jackson Laboratory. All the mice were C57BL/6 background and 8 to 10 weeks old. We used littermates with the same age and gender for each group. Animal use and protocols were approved by the Institutional Animal Care and Use Committees at the Institute of Biophysics, Chinese Academy of Sciences.

### Mouse treatment

For antibiotic (Abx) treatment, 8-week mice were fed water containing vancomycin (0.5 g/liter), ampicillin (1 g/liter), kanamycin (1 g/liter), and metronidazole (1 g/liter) for 2 weeks. Fresh Abx was replaced every other day. For microbiota metabolite treatment, metabolites were dissolved in drinking water. Mice were treated with 200 μl of indicated concentration of metabolites once a day by gavage administration at the beginning of antibiotic treatment. Treatment continued for 2 weeks.

### IBD sample collection and ethical approval

All patients are from The Affiliated Wuxi People’s Hospital of Nanjing Medical University. The patients were treatment naive and included upon diagnostic colonoscopy. IBD colitis diagnosis was endoscopically and histologically verified. Biopsies were obtained from two areas in the ileum of patients with IBD: the endoscopically most affected area (inflamed) and the endoscopically least-affected area (noninflamed). All participants provided written informed consent to sample collection and data analysis. This study was approved by the Research Ethics Committee at The Affiliated Wuxi People’s Hospital of Nanjing Medical University.

### Parabiosis surgery

Female 8-week-old congenic CD45.1 and CD45.2 mice were surgically connected in parabiosis according to a published protocol (*52*). In brief, the mice were first anesthetized with tribromoethanol (300 mg/kg). Then, the skin along a continuous line from the elbow to the knee on the side to be jointed was shaved and sterilized with iodine and alcohol. After corresponding lateral skin incisions were made from elbow to knee in each mouse, forelimbs and hindlimbs were tied together using nylon suture, and the skin incisions were closed using stainless steel wound clips. The mice were placed on a heated pad in a supine position and allowed to wake up in ambient air. After the surgery, each pair of mice was housed separately, and subcutaneous injections of 0.25% bupivacaine (2.5 mg/ml, 8 mg/kg) were given daily for 3 days for pain management. The mice remained surgically jointed for at least 2 months. T cell and B cell equilibration in the spleen and blood was confirmed by assessing presence of CD45.1/CD45.2 congenic markers. Last, the parabionts were euthanized, and tissues from CD45.1^+^ parabionts were processed and analyzed by flow cytometry for the presence of CD45.2^+^ ILC subsets.

### Isolation of mononuclear cells

BM cells were flushed out from femurs in staining buffer [PBS buffer containing 1% fetal bovine serum (FBS)] and filtered through 40-μm cell strainers. Spleens and mLN were physically dissociated using ground glass slides and filtered through a 40-μm strainer. Red blood cells in spleen were lysed using red lysis buffer. The SI was dissected, and the fat and PPs were removed. Next, the SI was open up lengthwise, and the lumen contents were washed out. Then, SI tissue was cut into 0.5 cm-long pieces, placed in solution I buffer [10 mM Hepes and 5 mM EDTA in Hanks’ balanced salt solution (HBSS), pH 7.2] to incubate three times for 15 min each at 37°C. The cell suspension was collected and centrifuged after three rounds of digestion. Density gradient step was done by resuspending cells in 44% Percoll and carefully laid on 67% Percoll solution and centrifuged at 600*g* and room temperature for 20 min. Intraepithelial lymphocytes (IELs) were at the 44 to 67% interface. For lamina propria lymphocyte (LPL) isolation, remaining intestinal fragments were cut into fine pieces and digested three times for 20 min each at 37°C with solution II buffer [2% FBS and collagenase II and collagenase III (0.5 mg/ml)], and, then, cell suspension was collected after three rounds of digestion. Collected IELs and LPLs were filtered through 40-μm strainers before being centrifuged and resuspended in an appropriate amount of staining buffer. For cell isolation from ileac biopsies, biopsies were cut into pieces and digested three times for 20 min each at 37°C with digestion buffer [2% FBS and collagenase II and collagenase III (0.5 mg/ml)], and, then, cell suspension was collected and filtered through 40-μm strainers. After centrifugation, the cells were resuspended in staining buffer.

### Flow cytometry

For flow cytometry, cells were isolated and stained with antibodies targeting surface antigens for 30 to 40 min on ice, followed by washing with staining buffer (PBS containing 1% FBS). For intracellular staining, cells were harvested after surface marker staining, then fixed, and permeabilized with the FoxP3 Transcription Factor Staining Kit (Thermo Fisher Scientific) according to the manufacturer’s instructions. Antibodies for intracellular staining were incubated with cells on ice for 30 to 40 min in 1× permeabilization buffer. For flow cytometry analysis, ILCs (Lin^−^Thy1.2^+^CD127^+^), ILC1s (Lin^−^CD127^+^CD45.2^+^NK1.1^+^NKp46^+^), ILC2s (Lin^−^CD127^+^Thy1.2^+^KLRG1^+^Sca-1^+^), ILC3s (Lin^−^CD127^+^Thy1.2^+^RORγt^+^), common lymphoid progenitor (CLP, Lin^−^CD127^+^c-Kit^lo^Sca-1^lo^Flt3^+^α4β7^−^), common ILC progenitor (CILP, Lin^−^CD25^−^CD127^+^Flt3^−^α4β7^+^), common helper-like ILC progenitor (CHILP, Lin^−^CD25^−^CD127^+^Flt3^−^α4β7^+^Id2^+^), and ILC progenitor (ILCP, Lin^−^CD127^+^Flt3^−^α4β7^+^c-kit^+^PLZF^+^) were analyzed with a FACSAria III instrument (BD Biosciences). Results were analyzed by FlowJo 10.

### Quantitative real-time PCR

For analysis of sorted cell populations, total RNA was extracted through the RNA Miniprep Kit (Tiangen, Beijing, China) following the manufacturer’s instructions. For analysis of SI tissues and IBD samples, tissues or samples were homogenized directly into TRIzol (QIAGEN), and RNA was extracted via chloroform extraction. The quantity and quality of RNA were determined by using a NanoDrop (Thermo Fisher Scientific). For both methods, cDNA was synthesized with 5× All-In-one RT MasterMix (Abm, Vancouver, Canada) and analyzed on QuantStudio1 qPCR system using specific primer pairs listed in table S2. Relative expression was calculated and normalized to endogenous *Actb*.

### Immunofluorescence staining

SIs and colons were cut open longitudinally and rinsed with precold 1× PBS. The tissues were then rolled into a “Swiss roll” shape and fixed in 4% PFA for 1 hour at room temperature. After fixation, the samples were dehydrated in a 30% sucrose solution for 24 hours and then embedded in optimal cutting temperature (OCT) freezing medium for sectioning. For immunofluorescence staining, the tissue sections were briefly rehydrated in PBS and then permeabilized with 1% Triton X-100 solution (PBS diluted) for 1 hour, followed with blocking in 10% donkey serum (diluted in PBS) for 30 min. The blocked sections were incubated with prediluted primary antibodies overnight at 4°C on a shaker. After washing with PBST (0.05% Tween 20 in PBS) three times, 5 min each time, sections were incubated with secondary antibodies for 1 hour at room temperature and then washed with PBST. Last, stained sections were mounted with Fluormount G (SouthernBiotech) and examined on a Nikon laser scanning confocal microscope. Images were analyzed by Imaris 9 software.

### ILC2s’ in vitro stimulation

Sort-purified small intestinal ILC2s were incubated in complete RPMI 1640 medium [containing 10% FBS, 50 μM 2-mercaptoethanol, 1 mM l-glutamine, 1 mM sodium pyruvate, penicillin (100 U/ml), and streptomycin (100 μg/ml)] for 24 hours at 37°C and 5% CO_2_ with IL-2 (10 ng/ml) and IL-7 (10 ng/ml). IL-25 (50 ng/ml) and IL-33 (50 ng/ml) were added if indicated. For intracellular cytokine staining, the culture was supplemented with brefeldin A (10 μg/ml) for 4 hours.

### In vivo antibody labeling

PE-conjugated anti-CD45.2 antibody (2 μg) was intravenously injected (intravenously) into mice, which had been pretreated with antibiotics for 2 weeks. Ten minutes later, mice were euthanized, and SI was harvested to isolate mononuclear cells for further flow cytometry analysis.

### Intestinal homing of ILCs

To analyze short-term homing of ILCs, intestinal lymphocytes were isolated from CD45.2^+^ WT mice as described above, and, then, Lin^−^ lymphocytes were sorted. Sorted CD45.2^+^ Lin^−^ lymphocytes (1 × 10^7^) were transplanted into lethally irradiated (2 × 5 gray with a 4-hour interval) recipient mice (CD45.1^+^) pretreated with or without antibiotics for 2 weeks. The recipients were euthanized 18 hours later, and SI was harvested and examined by flow cytometry for frequencies of transferred ILCs.

### 3D fluorescence imaging

3D fluorescence imaging was performed by using a slightly modified protocol previously published (*53*). In brief, mice were perfused with cold PBS, followed by 4% PFA. Then, strips of SI were pinned onto agarose plates and fixed overnight with 4% PFA in PBS. After several rinses in PBS, samples were passed through a glycerol and sucrose gradient, permeabilized, and blocked in permeabilizing solution [0.1% Tween 20, 0.5% Triton X-100, 0.1% deoxycholate, 0.1% NP-40, and 10% bovine serum albumin (BSA) in PBS] at 4°C for 1 day with gentle shaking. An appropriate primary antibody mixture in blocking buffer (0.5 Triton X-100 and 3% BSA in PBS) was performed on samples and incubated for 1 day on a shaker at 4°C. After washing in washing buffer (1% Triton X-100 in PBS) for 5 hours with a change of buffer every 30 min on a shaker at 4°C, secondary antibodies were diluted in blocking buffer and performed on samples for 1 day on a shaker at 4°C, and, then, samples were washed in washing buffer as mentioned above. For confocal imaging, sample was properly sliced to 1-mm fragments with blades and placed on glass slides preadded with mounting medium Fluormount G (SouthernBiotech) and then covered by a coverslip. Samples were imaged by using confocal scanning (Nikon), and *z*-stacks projections were compiled by using Imaris 9 software.

### Ca^2+^ mobilization assay

*P2ry10* sequence was cloned into pCDNA4 plasmid for overexpression. Gα12, Gα13, Gα15, and Gα16 sequences were cloned into p3 × FLAG-CMV plasmid. Promiscuous expression vectors were cotransfected into HEK293T cells using Lipofectamine 3000 (Invitrogen) according to the manufacturer’s instructions. The transfected cells were cultured for 24 hours at 37°C and then were collected and seeded in fibronectin (Sigma-Aldrich)–coated 96-well plates. After continuing to culture for 16 hours, cells were washed with washing buffer [1× HBSS with Ca^2+^/Mg^2+^, 10 mM Hepes, and 0.1% BSA (pH 7.4)]. Then, cells were loaded with 2 μM Ca^2+^ indicator (Fluo-4 AM) diluted with washing buffer supplemented with 0.05% pluronic-F127 (Sigma-Aldrich) and 1 mM probenecid (MCE) and then cultured in 37°C incubator for 30 min. Later, loading buffer with indicator was replaced with washing buffer to incubate for another 30 min, and, then, cells were washed again and loaded with washing buffer. Intracellular Ca^2+^ flux induced by stimulation of different metabolites was analyzed by Nikon confocal through collecting fluorescence images at one frame per second after the metabolite was added into cells. Collection process was lasted for 180 s, and, then, the fluorescence intensity of every image was analyzed by NIS Elements AR software, and fluorescence/background (*F*/*F*_0_) was quantified over time as an index of P2Y10 receptor activation.

### Analysis of activated RhoA signaling

Active RhoA (guanosine 5′-triphosphate bound) was determined by RhoA G-LISA activation kits (Cytoskeleton). HEK293T cells overexpressing P2Y10 and combined G proteins were obtained as above and then stimulated by indicated metabolite. Basal levels and metabolite-induced changes in activation were determined.

For luciferase reporter assay to determine the activation of RhoA signaling, HEK293T cells overexpressing P2Y10 and combined G proteins were seeded into six-well plates. Subsequently, 2 μg of pGL4.34[luc2P/SRF-RE/Hygro] plasmid (ZHUANGMO Bioscience, ZK39238) was transfected into the cells, which were then collected after 6 hours and seeded into 96-well plates. Following a 12-hour incubation, the culture medium was replaced with serum-free Dulbecco’s modified Eagle’s medium (DMEM) (100 μl per well) and continued to incubate for 8 hours. To stimulate the cells, the metabolites with indicated concentrations were diluted in DMEM containing 10% FBS and added to each well after removing 50 μl of the original culture medium. The cells were then incubated at 37°C for 6 hours. A control group without stimulation was set up. The relative light units (RLU) of luminescence signals were detected with the ONE-Glo Luciferase Assay System (Promega, E6110) according to the manufacturer’s protocol. Fold induction = RLU_induced_/RLU_noninduced_.

### Analysis of activated RhoA signaling in ILCs

To analyze the active RhoA levels in intestinal ILCs by flow cytometry, LPLs were isolated and stained with specific antibodies against lineage, Thy1.2, and CD127. After washing, the cells were stimulated with 10 μM TDCA for 5 min. Subsequently, the cells were fixed with 4% PFA and permeabilized with 0.1% Triton X-100 for 30 min on ice. The cells were then incubated with glutathione *S*-transferase (GST)–tagged Rho binding domain (RBD) of rhotekin for 3 hours at room temperature, followed by washing with TBST and incubation with rabbit anti-GST antibodies (no. ab111947, Abcam; 1:200) for 2 hours at room temperature. After washing, the cells were incubated with Alexa Fluor 488–labeled goat anti-rabbit secondary antibodies for 1 hour at room temperature. The flow cytometry analysis was performed to determine the active RhoA levels in ILCs.

To analyze the intracellular distribution of active RhoA using immunofluorescence, total ILCs were sorted from the SI of *P2ry10*^+/+^ and *P2ry10*^−/−^ mice. The sorted cells were seeded on chambered coverslips coated with 10% poly-l-lysine solution. The cells were then stimulated with 10 μM TDCA for 5 min. After fixation with 4% PFA and permeabilization with 0.1% Triton X-100 for 10 min at 4°C, the cells were blocked with 10% donkey serum in PBS at room temperature. Subsequently, the cells were incubated with GST-tagged RBD of rhotekin for 24 hours at 4°C, then washed, and incubated with rabbit anti-GST antibodies overnight at 4°C. After washing, the cells were incubated with Alexa Fluor 488–labeled goat anti-rabbit secondary antibodies for 1 hour at room temperature. Last, the cells were stained with DAPI and imaged by using a Nikon confocal microscope.

### DSS-induced inflammation model

*P2ry10*^+/+^ and *P2ry10*^−/−^ mice were treated with 2% (w/v) DSS (MP Biomedicals) in the drinking water for 5 days and followed by regular drinking water for 5 days. For TDCA-treated mice, mice were treated with TDCA (1 mg/20 g of body weight, diluted in drinking water) via orally gavage daily for 2 weeks while maintained on Abx drinking for 2 weeks, and, then, mice with different treatment were treated with 2% (w/v) DSS as above. Body weight was measured every day and mice were euthanized at day 10. Colons were collected for pathological analysis.

### BM transductions and transplantation

For *Zfp414* knockout in BM cells, sg*Zfp414s* (table S1) were cloned into the adeno-associated virus (AAV) vector with Cre recombinase. sgRNA sequences were selected by using Benchling’s CRISPR Guide tool. HEK293T cells were used for adenovirus packaging. AAV plasmid (8 8 μg), packaging plasmid pHelper (4 μg), and pAnc80L65 (4 μg) were mixed and transfected into HEK293T cells by using Lipofectamine 3000 reagent. On the second day after transfection, intracellular AAV was collected by freeze thawing with liquid nitrogen and then concentrated via ultracentrifuged at 25,000 rpm for 2 hours at 4°C. For *Zfp414* overexpression, the CDS sequence of *Zfp414* was cloned into PLVX-IERS-Puro lentivirus vector. Concentrated lentivirus was obtained as above. For BM transduction, Rosa26-cas9-P2A-EGFP mice were injected intravenously with 3 mg of 5-fluorouracil (Sigma-Aldrich). After 4 to 5 days, BM was collected and cultured in complete RPMI 1640 medium supplemented with IL-3 (20 ng/ml), IL-6 (50 ng/ml), and SCF (100 ng/ml). BM cells were spin infected (1000*g*, 2 hours, 32°C) at days 1 and 2 to introduce viral vectors. After the second infection, the infected BM cells (5 × 10^6^) were 1:1 mixed with BM cells from CD45.1^+^ mice. The mixed cells were then transplanted into lethally irradiated CD45.1^+^ recipients. Two months later, flow cytometry analysis was performed.

### In vitro differentiation assay and ILCs labeling

Intestinal ILC in vitro differentiation assay was performed as previously described (*54*). Briefly, cells from femurs were flushed out by using PBS containing 1% FBS and filtered through 40-μm strainers. Collected cells were treated with red blood cell lysis buffer (Tiangen, Beijing) to exclude red cells. CHILPs (Lin^−^CD25^−^CD127^+^Flt3^−^α4β7^+^Id2^GFP^) and ILCPs (Lin^−^CD127^+^Flt3^−^α4β7^+^c-Kit^+^PLZF^GFP^) were sorted by FACSAria III instrument and transduced by lentivirus containing sh*Itgae* through spin infection (1000*g*, 2 hours, 32°C) at days 1 and 2. OP9-DL1 cells were maintained in complete minimum essential medium alpha (αMEM) medium (supplemented with 10% FBS, 1% streptomycin, and 1% penicillin). Before seeding of progenitor cells, OP9-DL1 cells were treated with mitomycin (4 μg/ml) for 2 hours to inhibit cell division. Then, cells were digested and seeded at the density of 1 × 10^6^ cells per well in a 24-well plate. After OP9-DL1 cells were adhered, and indicated progenitor cells were inoculated on OP9- DL1 feeder cells in a complete RPMI 1640 medium [supplemented with 10% FBS, 1% streptomycin, 1% penicillin, IL-7 (20 ng/ml), and SCF (20 ng/ml)]. After 14 days, differentiated ILCs were harvested and labeled with carboxyfluorescein diacetate succinimidyl ester (CFSE; 5 μM; Thermo Fisher Scientific, C34570) for 15 min at 37°C, and, then, CFSE was quenched with PBS for 3 min. Labeling ILCs (5 × 10^6^) were resuspended with complete RPMI 1640 medium and injected intravenously into immunodeficient NSG mice. The recipients were euthanized 18 hours later for further immunofluorescence.

### *C. rodentium* infection

*C. rodentium* was a gift from B. Ge (Shanghai Institutes for Biological Sciences, Chinese Academy of Sciences). Mice were fasted for 5 hours and then orally infected with 5 × 10^9^ colony-forming units (CFU) of *C. rodentium*. Mice were weighed daily; on day 10 after infection, mice were euthanized, and colons were collected for pathological analysis. Feces were collected and homogenized for bacterial load measurement.

### Histopathological analysis

Colons from indicated mice were cut open longitudinally and rinsed with PBS before fixation in 4% PFA followed by paraffin sectioning and hematoxylin and eosin (H&E) staining. The histopathological score was evaluated and analyzed according to a previous study (*55*). Briefly, the sections were evaluated for leukocyte infiltration (0 for absent, 1 for mild, 2 for moderate, and 3 for severe infiltration), epithelial injury (0 for normal villus structure, 1 for mild epithelium loss, 2 for moderate epithelium loss, and 3 for severe epithelium loss), submucosal edema (0 for no edema, 1 for <50% submucosal area and <100 mm wide, 2 for 50 to 80% submucosal area and <200 mm wide, and 3 for >80% submucosal area and > 200 mm wide), and the number of goblet cells (0 for >40 goblet cells per visual field, 1 for 26 to 40 goblet cells per visual field, 2 for 16 to 25 goblet cells per visual field, and 3 for 0 to 15 goblet cells per visual field).

### Inhibition of RhoA and Ca^2+^ signaling cascade

Sorted intestinal ILCs were rested for 2 hours in RPMI 1640 medium at 37°C. Then, cells were incubated with 1 μM Ca^2+^ inhibitor U-73122 (HY-13419-5, MCE), 1 μM ROCK inhibitor (S)-H-1152, 1 μM (10007653, Cayman), or vehicle (dimethyl sulfoxide) during overnight stimulation with TDCA at 37°C. Then, the mRNA expression of *Cd69*, *S1pr1*, and *Itgae* was analyzed by qPCR.

### ChIP assay

Intestinal ILCs were isolated and crosslinked with 1% formaldehyde at 37°C for 10 min. After two PBS washes, cells were lysed with SDS-containing buffer and subjected to sonication to generate DNA fragments between 200 and 500 base pairs. Lysates underwent preclearance with Protein A/G magnetic beads before overnight incubation at 4°C with 2 μg of ZFP414 antibodies. Following immunoprecipitation and sequential washes, bound DNA complexes were eluted and purified for subsequent analysis. Quantitative detection was performed using qPCR with primer pairs detailed in table S3.

### Transcriptome and bioinformatic analysis

ILCs (Lin^−^Thy1.2^+^CD127^+^; 1 × 10^6^) from Ctrl and Abx-treated or *P2ry10*^+/+^ and *P2ry10*^−/−^ mice were sorted by using flow cytometry, and cells were lysed with TRIzol reagent, and, then, total RNA was extracted and subjected to RNA-seq by BGI Tech Company. R packages of “clusterProfiler” in Bioconductor were used for generating GO analysis. For bioinformatic analysis of RNA-seq of Ctrl and Abx-treated ILC1, ILC2s, and ILC3s, the scRNA-seq data were from GSE85154 (*25*).

### Liquid chromatography–mass spectrometry

For screening metabolites affected by microbiota deficiency, the contents of intestinal lumen were obtained and homogenized. Then, 400 μl of precooled methanol/acetonitrile/water solution (4:4:2, v/v/v) was added to 1 ml of sample. The mixture was vortexed, incubated at −20°C for 60 min, and then centrifuged at 14,000*g* for 20 min at 4°C. The supernatant was collected and dried in vacuum. For mass spectrometry (MS) analysis, 100 μl of acetonitrile-water solution (acetonitrile:water = 1:1, v/v) was added to reconstitute the samples. The mixture was vortexed and centrifuged at 14,000*g* for 15 min at 4°C, and 2 μl of the supernatant was taken for analysis. The chromatographic separation was performed by using an ACQUITY UPLC BEH C18 column (100 mm by 2.1 mm, 1.7 μm, Waters, USA) at a column temperature of 40°C and a flow rate of 0.3 ml/min. The mobile phase consisted of water with 0.1% formic acid as solvent A and acetonitrile as solvent B. The following gradient was used for metabolite elution: 0 to 0.5 min, 5% B; 0.5 to 1.0 min, 5% B; 1.0 to 9.0 min, 5 to 100% B; 9.0 to 12.0 min, 100% B; 12.0 to 15.0 min, 5% B. The injection volume for each sample was 5 μl. After separation by ultrahigh performance liquid chromatography (UHPLC), the samples were subjected to MS analysis using a Q-Exactive quadrupole-Orbitrap high-resolution mass spectrometer (Thermo Fisher Scientific). The analysis was operated in positive and negative electrospray ionization (ESI) mode. ESI settings were as follows: Ion Source Gas1, 60; Ion Source Gas2, 60; Curtain gas, 30; source temperature, 320°C; and IonSapary Voltage Floating, ±3500 V for both positive and negative ion modes. The MS scan mass/charge ratio range was set from 80 to 1200 Da, with a product ion scan resolution of 17,500. The MS scan accumulation time was 0.20 s per spectra, and the product ion scan accumulation time was 0.05 s per spectra. The second-stage MS was performed by using information-dependent acquisition in high-sensitivity mode. The following settings were used: exclude isotopes within 4 Da; candidate ions to monitor per cycle, 6. The declustering potential was set at ±60 V for both positive and negative ion modes, and the collision energy was set at 35 ± 15 eV. Data from liquid chromatography–MS/MS was analyzed with Compound Discoverer 3.0 (Thermo Fisher Scientific) and searched in BioCyc, HMDB, METLIN, HFMDB, and LIPID MAPS databases.

### Statistics and reproducibility

The numbers of mice and experiments, along with the statistical tests, are reported in each figure legend. For statistical evaluation, data were analyzed by GraphPad Prism 8.0. Two-tailed unpaired Student’s *t* test was used in this study. Results are shown as means ±SD. *P* values of ≤ 0.05 were considered significant (*P < 0.05; **P < 0.01; ***P < 0.001), and *P* > 0.05 was considered nonsignificant (n.s.). All flow cytometry data were analyzed by FlowJo 10.
