# Supplementary material for: Microbiota metabolite taurodeoxycholic acid maintains intestinal tissue residency of innate lymphoid cells via engagement with P2Y10 receptor
Source: Sci Adv. 2025 Aug 22;11(34):eadt9645. doi: 10.1126/sciadv.adt9645 (PMC12372891; doi:10.1126/sciadv.adt9645)
Supplement: Supplementary file 1 — Figs. S1 to S10 Tables S1 to S3 [file sciadv.adt9645_sm.pdf]

Science Advances
AAAS

Supplementary Materials for

Microbiota metabolite taurodeoxycholic acid maintains intestinal tissue residency of innate lymphoid cells via engagement with P2Y10 receptor

Yuwei Xu *et al.*

Corresponding author: Zusen Fan, fanz@moon.ibp.ac.cn; Yun Chen, chenyun@njmu.edu.cn;
Yong Tian, ytian@ibp.ac.cn

Sci. Adv. **11**, eadt9645 (2025)
DOI: 10.1126/sciadv.adt9645

This PDF file includes:

Figs. S1 to S10
Tables S1 to S3

Supplementary Figures and Tables

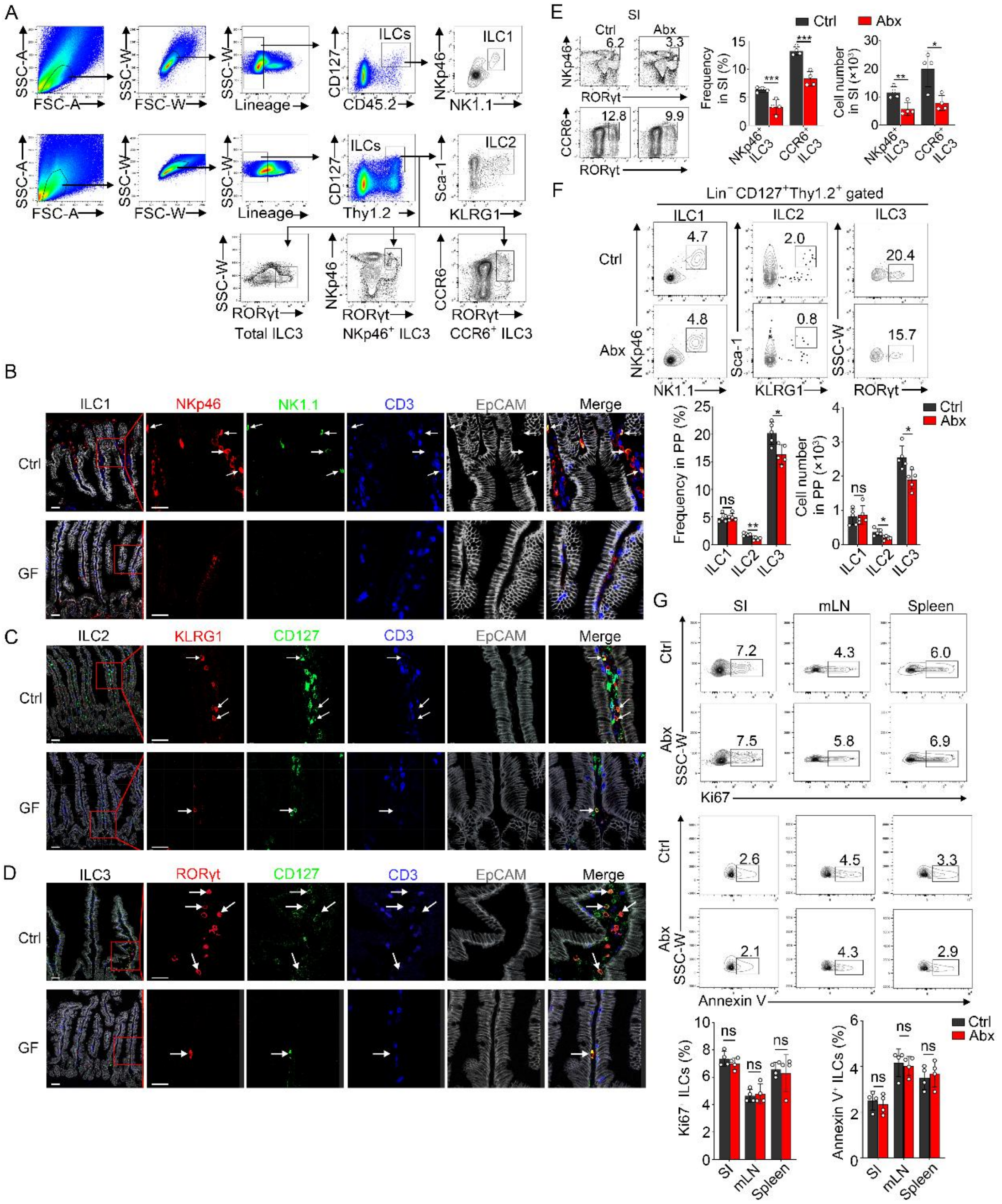

Figure S1. Microbiota disruption causes decreased numbers of resident ILCs in small intestine. (A) Gating strategy for total ILCs (Lin$^-$Thy1.2$^+$CD127$^+$), ILC1s (Lin$^-$CD127$^+$CD45.2$^+$NK1.1$^+$NKp46$^+$), ILC2s (Lin$^-$CD127$^+$Thy1.2$^+$KLRG1$^+$Sca-1$^+$), total ILC3s (Lin$^-$CD127$^+$Thy1.2$^+$RORγt$^+$), NKp46$^+$ ILC3s (Lin$^-$CD127$^+$Thy1.2$^+$RORγt$^+$ NKp46$^+$) and CCR6$^+$ ILC3s (Lin$^-$CD127$^+$Thy1.2$^+$RORγt$^+$CCR6$^+$). (B-D) Immunofluorescence staining of intestinal ILC1s (B), ILC2s (C) and ILC3s (D) from Ctrl and GF mice. White arrows denote indicated ILC subsets in the small intestine. Scale bar, 50 μm. (E) Flow cytometry analysis of NKp46$^+$ ILC3s (Lin$^-$CD127$^+$Thy1.2$^+$RORγt$^+$NKp46$^+$) and CCR6$^+$ ILC3s (Lin$^-$CD127$^+$Thy1.2$^+$RORγt$^+$CCR6$^+$) in the small intestine from Ctrl and Abx-treated mice which were fed water containing a combination of antibiotics (Abx) for two weeks.

Numbers in flow cytometry plots represent percentages of indicated ILC3 subsets. Frequencies and numbers of indicated ILC3 subsets are shown in right panel. n = 4 for each group. (F) Flow cytometry analysis of ILC1s, ILC2s and ILC3s from intestinal Peyer's patches (PP) in Ctrl and Abx mice. Numbers in flow cytometry plots represent percentage of indicated ILCs in each gate. Frequencies and numbers of indicated ILCs subsets are shown in lower panel. n = 5 for each group. (G) Flow cytometry analysis of $Ki67^+$ and Annexin V^+ ILCs in the small intestine from Ctrl and Abx-treated mice. Numbers in flow cytometry plots represent percentage of indicated ILCs in each gate. n = 4 for each group. Data are representative of at least three independent experiments and are shown as the means ± SD. Statistical analysis was performed by using unpaired two-tailed Student's t test (*P < 0.05, **P < 0.01, ***P < 0.001, ns, not significant).

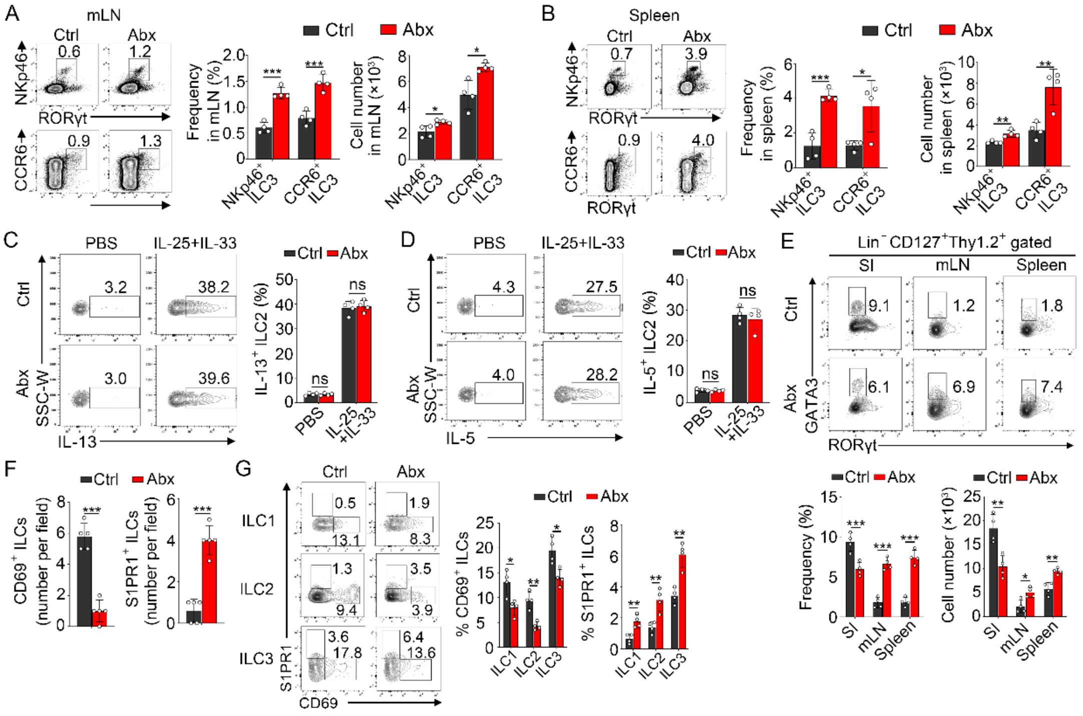

Figure S2. Microbiota disruption increases egression of intestinal ILCs. (A, B) Flow cytometry analysis of NKp46⁺ ILC3s and CCR6⁺ ILC3s in mLN (A) and spleen (B) from Ctrl and Abx-treated mice. Numbers in flow cytometry plots represent percentages of indicated ILC3 subsets. Frequencies and numbers of indicated ILC3 subsets are shown in right panel. n = 4 for each group. (C, D) Sort-purified small intestinal ILC2s from Ctrl and Abx-treated mice were cultured in vitro with or without IL-25 and IL-33. Flow cytometry analysis of IL-13⁺ (C) and IL-5⁺ ILC2s (D). Numbers in flow cytometry plots represent percentages of indicated ILC2s. Frequencies and numbers of indicated ILC2 are shown in right panel. n = 4 for each group. (E) Flow cytometry analysis of GATA3⁺ ILC2s (Lin⁻CD127⁺Thy1.2⁺GATA3⁺RORγt⁻) in the SI, mLN and spleen from Ctrl and Abx-treated mice. Numbers in flow cytometry plots represent percentages of ILC2s in each gate. Frequencies and numbers of ILC2s are shown in lower panel. n = 4 for each group. (F) Quantitative analysis of immunofluorescence results in Fig. 2A. The numbers of CD69⁺ and S1PR1⁺ ILCs per field are shown. n=5 fields for each group. (G) Flow cytometry analysis of CD69 and S1PR1 expression on ILC1s, ILC2s and ILC3s from Ctrl and Abx-treated mice. Numbers in flow cytometry plots represent percentages of indicated ILCs. Frequencies and numbers of indicated ILCs are shown in right panel. n = 4 for each group. Data are representative of at least three independent experiments and are shown as the means ± SD. Statistical analysis was performed by using unpaired two-tailed Student's t test (*P < 0.05, **P < 0.01, ***P < 0.001, ns, not significant).

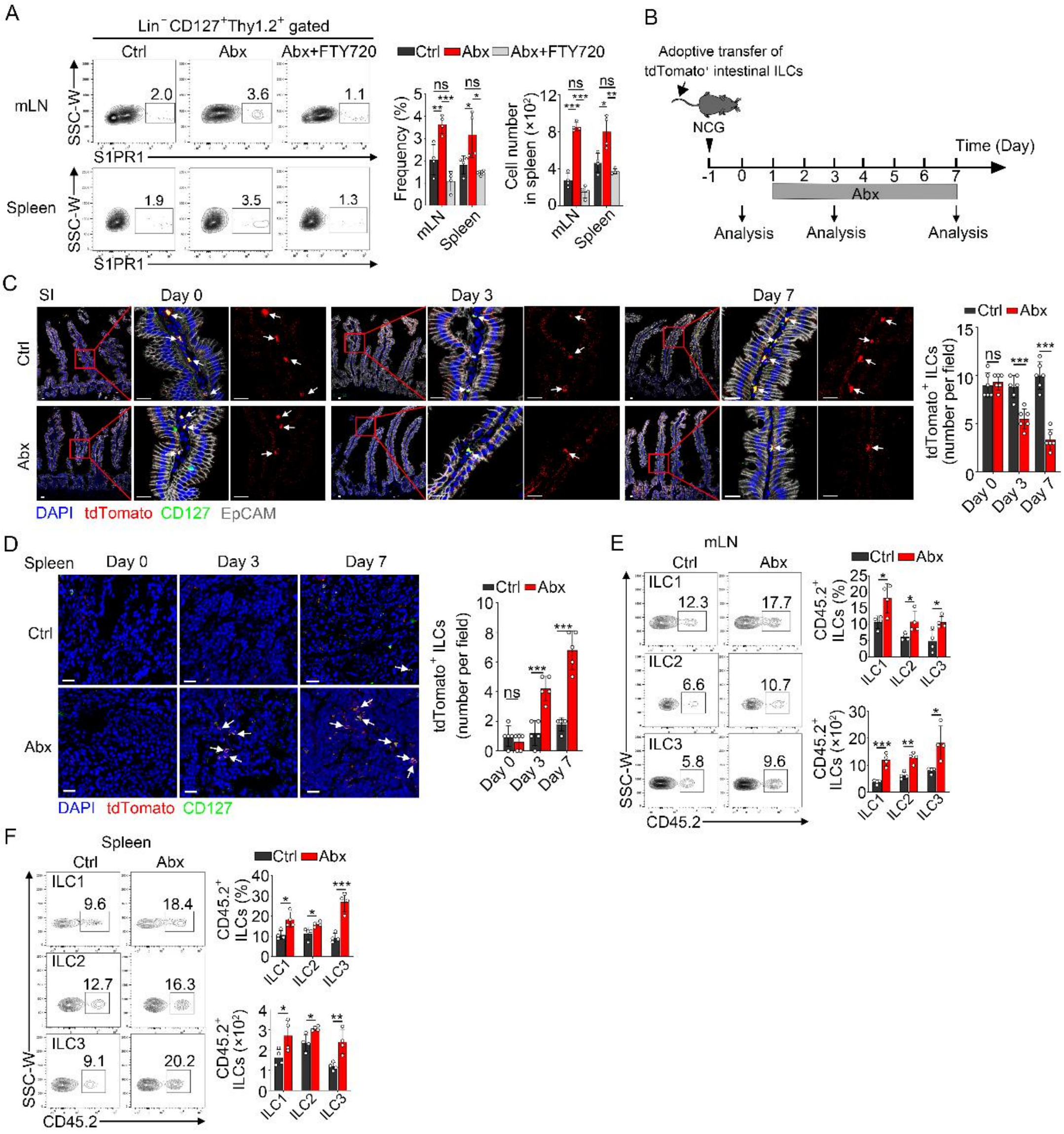

Figure S3. Microbiota disruption promotes migration of intestinal ILCs to the spleen and mLN. (A) Flow cytometry analysis of S1PR1⁺ ILCs in mLN and spleen from Ctrl, Abx-treated and Abx+FTY720 treated mice. Numbers in flow cytometry plots represent percentages of S1PR1⁺ ILCs. Frequencies and numbers of S1PR1⁺ ILCs are shown in right panel. n = 4 for each group. (B) Schema of adoptive transfer of tdTomato⁺ intestinal ILCs. At day -1, sorted tdTomato⁺ intestinal ILCs were transferred to immunodeficient mice. Then on day 1, the mice were treated with Abx for a week, and their intestine were collected on day 0, 3 and 7 for section analysis. (C, D) Immunofluorescence staining of tdTomato⁺ ILCs in small intestine (C) and spleen (D) from Ctrl and Abx-treated mice on day 0, 3 and 7. White arrows indicate tdTomato⁺ ILCs. Scale bar, 20 μm. The number of tdTomato⁺ ILCs per field are shown in right panel. n=6 fields for each group in (C). n = 5 fields for each group in (D). (E, F) 5×10^6 bone marrow (BM) cells from CD45.2⁺ donor mice were isolated and i.v. injected into lethally irradiated CD45.1⁺ Ctrl or Abx-treated recipients. Eight weeks later, flow cytometry analysis of donor-derived CD45.2⁺ ILC subsets in the mLN (E) and spleen (F). Numbers in flow cytometry plots represent

percentage of donor derived CD45.2$^+$ ILC subsets in each tissue. Frequencies and numbers of intestinal CD45.2$^+$ ILC subsets are showed in right panel. n = 4 for each group. Data are representative of at least three independent experiments and are shown as the means ± SD. Statistical analysis was performed by using unpaired two-tailed Student's t test (*P < 0.05, **P < 0.01, ***P < 0.001, ns, not significant).

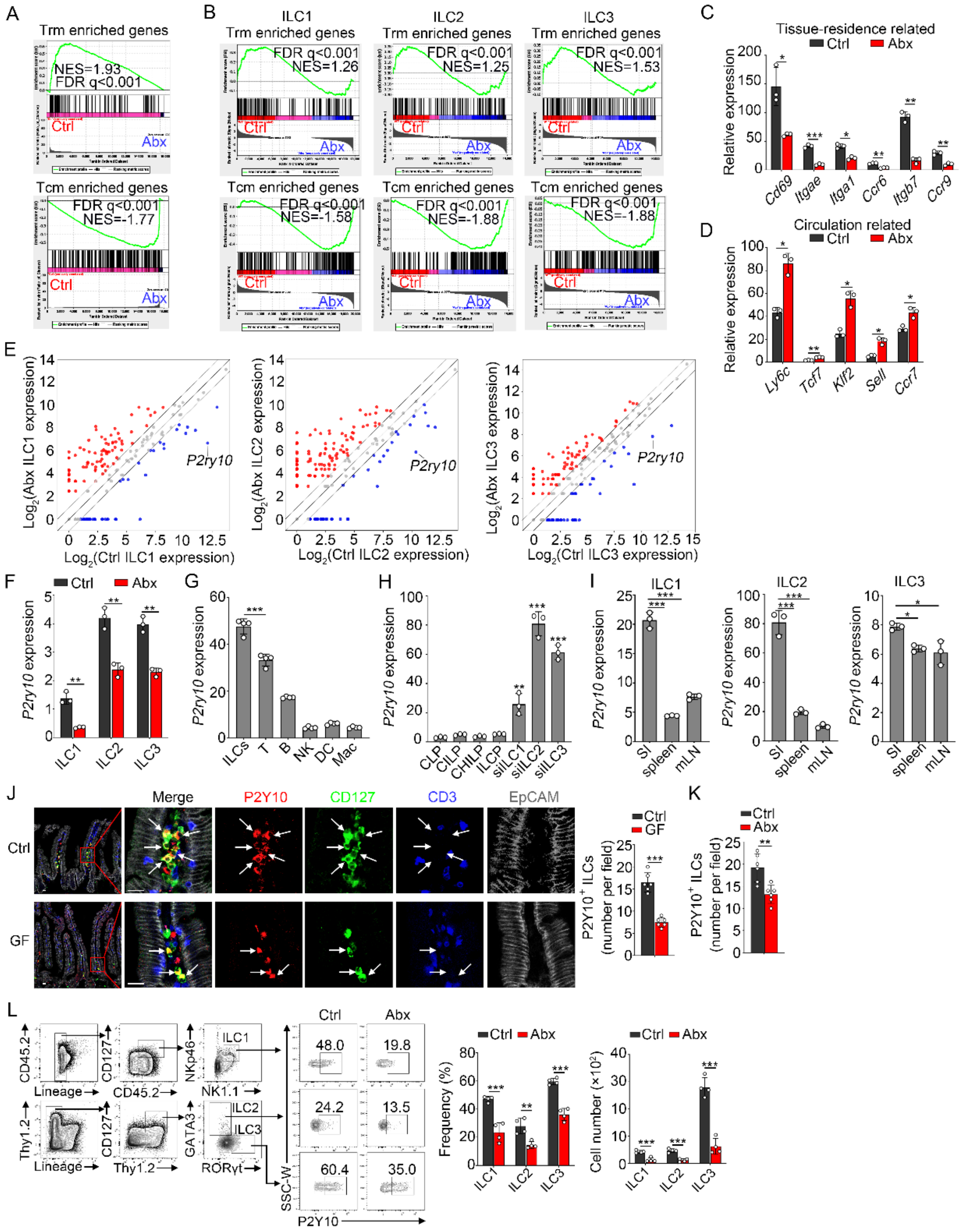

Figure S4. Intestinal ILCs highly express P2Y10 and genes related to tissue residency. (A, B) Gene set enrichment analysis (GSEA) for genes associated with tissue residency (genes are upregulated in Trm) or circulation (genes are upregulated in Tcm) in intestinal total ILCs (A), ILC1s, ILC2s and ILC3s (B) from Ctrl and Abx-treated mice. FDR, false discovery rate. NES, normalized enrichment score. (C, D) Relative expression levels of genes related to tissue residency (C) and circulation (D) in Ctrl or Abx ILCs were analyzed by qPCR. Fold changes were normalized to *Actb*. (E) Expression of GPCRs in ILC1s, ILC2s and ILC3s from Ctrl and Abx-treated mice are shown, and the expression of *P2ry10* is marked. Data were from Gury-BenAri's scRNA-seq datasets (GSE85154). (F) Relative mRNA levels of *P2ry10* in Ctrl or Abx-treated ILC1s, ILC2s and ILC3s were

analyzed by qPCR. (G) Relative expression of *P2ry10* was measured in indicated intestinal leukocytes by qPCR. Fold changes were normalized to endogenous *Actb*. ILCs ($Lin^{-}CD127^{+}Thy1.2^{+}$), T cells ($CD3^{+}CD45^{+}CD19^{-}NK1.1^{-}$), B cells ($CD3^{-}CD45^{+}CD19^{+}NK1.1^{-}$), NK cells ($CD3^{-}CD45^{+}CD19^{-}NK1.1^{+}$), DC cells ($CD45^{+}CD11c^{+}CD11b^{-}F4/80^{-}$) and macrophage ($CD45^{+}CD11c^{-}CD11b^{+}F4/80^{+}$) were isolated from small intestine. (H) ILC progenitors and mature ILC subsets were sorted and the relative expression of *P2ry10* was measured by qPCR. CLP ($Lin^{-}CD127^{+}c\text{-}Kit^{lo}Sca\text{-}1^{lo}Flt3^{+}\alpha4\beta7^{-}$), CILP ($Lin^{-}CD25^{-}CD127^{+}Flt3^{-}\alpha4\beta7^{+}$), CHILP ($Lin^{-}CD25^{-}CD127^{+}Flt3^{-}\alpha4\beta7^{+}Id2^{GFP}$), and ILCP ($Lin^{-}CD127^{+}Flt3^{-}\alpha4\beta7^{+}c\text{-}kit^{+}PLZF^{GFP}$) were isolated from BM cells. siILC1s ($Lin^{-}CD45.2^{+}CD127^{+}NK1.1^{+}NKp46^{+}$), siILC2s ($Lin^{-}Thy1.2^{+}CD127^{+}KLRG1^{+}Sca\text{-}1^{+}$) and siILC3s ($Lin^{-}CD127^{+}CD45^{lo}$) were isolated from the small intestine. (I) Relative expression of *P2ry10* in ILC1s, ILC2s and ILC3s isolated from small intestine, mLN and spleen separately. Fold changes were normalized to *Actb*. (J) Immunofluorescence staining of ILCs expressing P2Y10 from Ctrl and GF mice with P2Y10 (red), CD127 (green), CD3 (blue) and EpCAM (grey) antibodies. White arrows indicate P2Y10+ ILCs in the small intestine. Scale bar, 20 μm. Numbers of $P2Y10^{+}$ ILCs per filed are shown in right panel. n=6 fields for each group. (K) Quantitative analysis of immunofluorescence results in Fig. 3D. Numbers of $P2Y10^{+}$ ILCs per filed are shown. n=6 fields for each group. (L) Flow cytometry analysis of P2Y10 expression on intestinal ILC1s, ILC2s and ILC3s from Ctrl and Abx-treated mice. Numbers in flow cytometry plots represent percentages of indicated ILC subsets. Frequencies and numbers of $P2Y10^{+}$ ILC subsets are shown in right panel. n = 4 for each group. Data are representative of at least three independent experiments and are shown as the means ± SD. Statistical analysis was performed by using unpaired two-tailed Student's t test (*$P < 0.05$, **$P < 0.01$, ***$P < 0.001$).

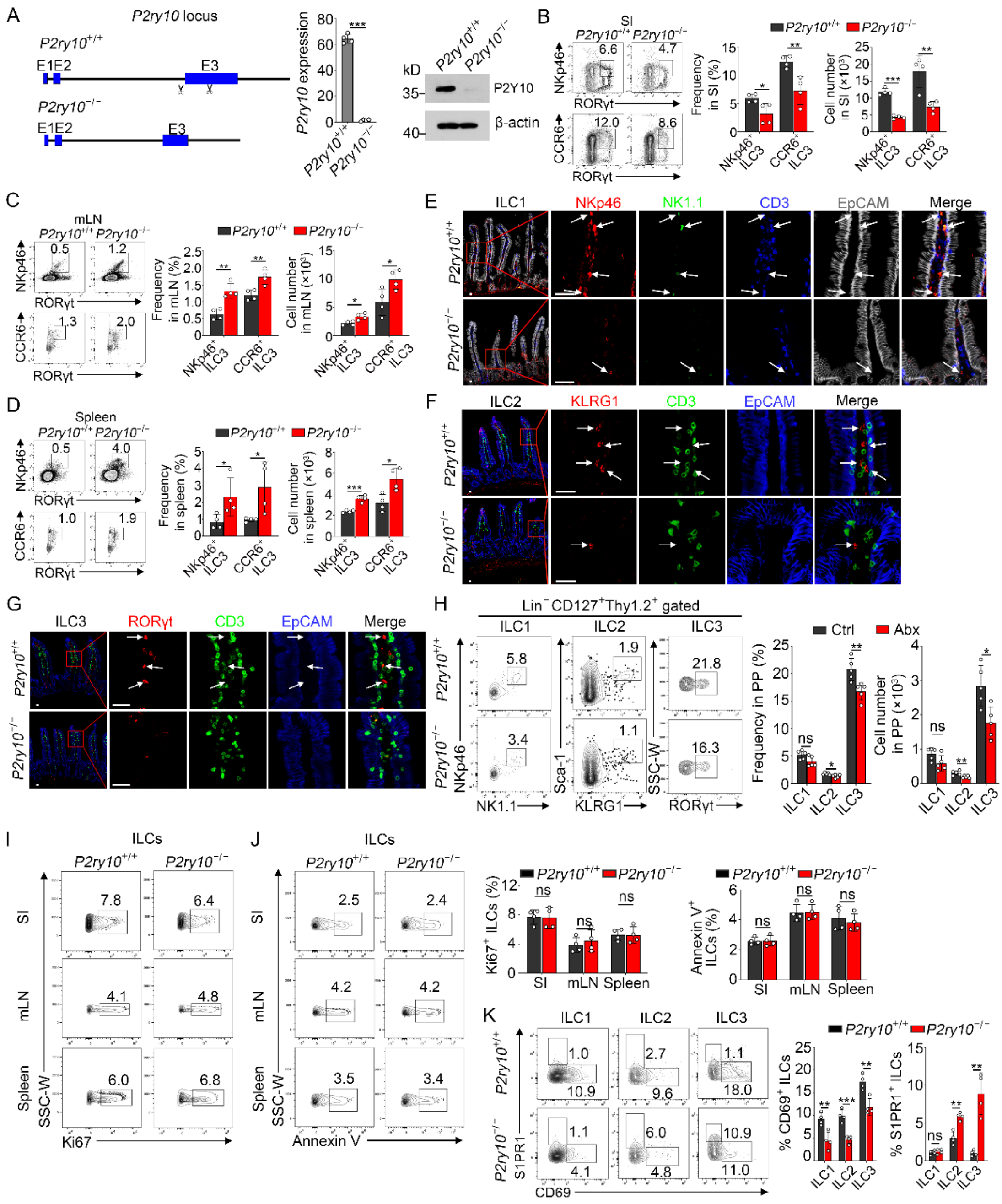

Figure S5. P2Y10 receptor is required for tissue residency maintenance of intestinal ILCs. (A) Schema for generation of *P2ry10*$^{-/-}$ mice using CRISPR/Cas9 technology. *P2ry10* deletion was validated by qPCR and western blot separately. Fold changes were normalized to *Actb*. (B-D) Flow cytometry analysis of NKp46^{+} ILC3s and CCR6^{+} ILC3s in the small intestine (B), mLN (C) and spleen (D) from *P2ry10*$^{+/+}$ and *P2ry10*$^{-/-}$ mice. n = 4 for each group. (E-G) Immunofluorescence staining of intestinal ILC1s (E), ILC2s (F) and ILC3s (G) from *P2ry10*$^{+/+}$ and *P2ry10*$^{-/-}$ mice. White arrows denote indicated ILC subsets in the small intestine. Scale bar, 20 μm. (H) Flow cytometry analysis of ILC1s, ILC2s and ILC3s from intestinal Peyer's patches (PP) in *P2ry10*$^{+/+}$ and *P2ry10*$^{-/-}$ mice. Numbers in flow cytometry plots represent percentages of indicated ILCs subsets. Frequencies and numbers of indicated ILCs subsets are shown in right panel. n = 4 for each group. (I, J)

Flow cytometry analysis of Ki67^{+} (I) and Annexin V^{+} (J) ILCs in the small intestine from *P2ry10*$^{+/+}$ and *P2ry10*$^{-/-}$ mice. Numbers in flow cytometry plots represent percentage of indicated ILCs in each gate. Frequencies of indicated ILCs subsets are shown in right panel. n = 4 for each group. (K) Flow cytometry analysis of S1PR1 and CD69 expression on ILC1s, ILC2s and ILC3s from *P2ry10*$^{+/+}$ and *P2ry10*$^{-/-}$ mice. Numbers in flow cytometry plots represent percentages of indicated ILC subsets. Frequencies and numbers of indicated ILC subsets are shown in right panel. n = 4 for each group. Data are representative of at least three independent experiments and are shown as the means ± SD. Statistical analysis was performed by using unpaired two-tailed Student's t test (*P < 0.05, **P < 0.01, ***P < 0.001, ns, not significant).

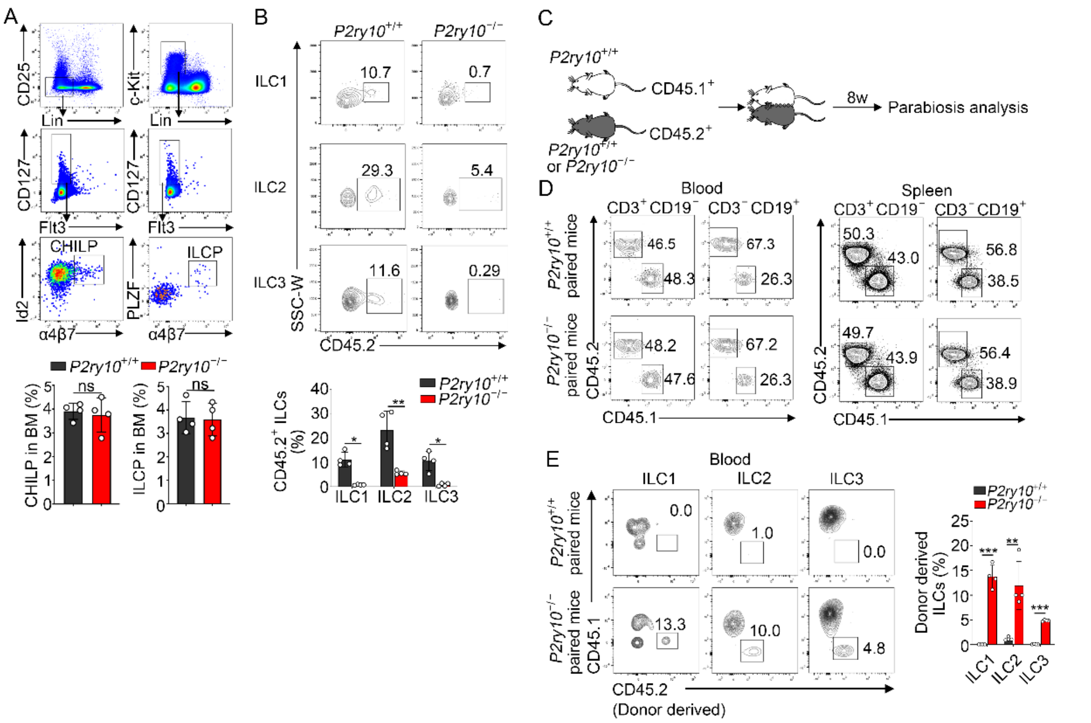

Figure S6. P2Y10 is required for intestinal homing and positioning of ILCs. (A) Flow cytometry analysis of CHILP and ILCP in BM cells of *P2ry10*$^{+/+}$ and *P2ry10*$^{-/-}$ mice. Numbers in flow cytometry plots represent percentage of indicated cells in each gate. n = 4 for each group. (B) 5×10^6 BM cells from *P2ry10*$^{+/+}$ or *P2ry10*$^{-/-}$ CD45.2$^+$ donor mice were 1:1 mixed with BM cells from CD45.1$^+$ mice. The mixed cells were then transplanted into lethally irradiated CD45.1$^+$ recipients. 2 months later, donor derived ILC subsets homing to intestine and long-term positioning there were analyzed by flow cytometry. Numbers in flow cytometry plots represent percentage of donor derived ILC subsets in each gate. Frequencies of CD45.2$^+$ ILC subsets in corresponding ILC subsets are showed in lower panel. n = 4 for each group. (C, D) *P2ry10*$^{+/+}$ or *P2ry10*$^{-/-}$ CD45.2$^+$ donor mice underwent parabiosis surgery with CD45.1$^+$ recipient mice. After 8 weeks, donor-derived T cell (CD3$^+$CD19$^-$) and B cell (CD3$^-$CD19$^+$) in the blood and spleen were analyzed by flow cytometry. (E) Flow cytometry analysis of donor-derived ILC1s, ILC2s and ILC3s in blood from CD45.1$^+$ recipient parabionts. Frequencies of donor-derived CD45.2$^+$ ILC1s, ILC2s and ILC3s in blood are showed in right panel. n = 4 for each group. Data are representative of at least three independent experiments and are shown as the means ± SD. Statistical analysis was performed by using unpaired two-tailed Student's t test (*P < 0.05, **P < 0.01, ***P < 0.001, ns, not significant).

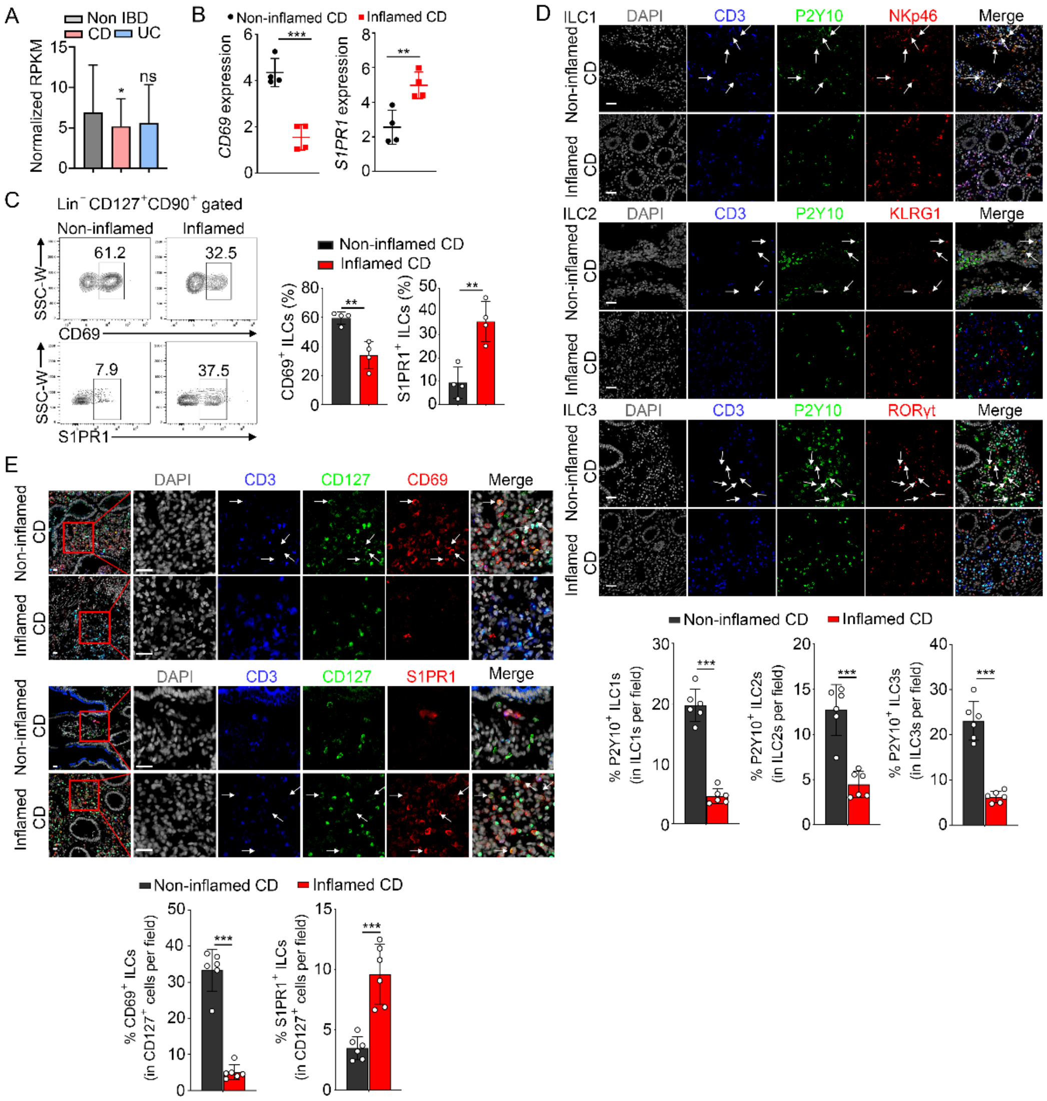

Figure S7. P2Y10 and CD69 are highly expressed on human ILCs from non-inflamed intestine. (A) The sequencing data of intestinal samples from non-IBD patients and patients with Crohn's disease (CD) and ulcerative colitis (UC) were obtained. The expression levels of *P2RY10* were analyzed. (B) Relative mRNA levels of *CD69* and *S1PR1* in ILCs from non-inflamed and inflamed intestinal samples of CD patients were analyzed by qPCR. Fold changes were normalized to *ACTB*. n = 4 independent experiments. (C) Lymphocytes were isolated from inflamed and non-inflamed intestinal samples of CD patients, and the expression of CD69 (n = 4) and S1PR1 (n = 4) in intestinal ILCs (Lin$^-$CD90$^+$CD127$^+$) was analyzed by flow cytometry. Numbers in flow cytometry plots represent percentage of CD69$^+$ and S1PR1$^+$ ILCs in each gate. Frequencies of CD69$^+$ and S1PR1$^+$ ILCs from different samples are shown in right panel. (D) Immunofluorescence staining of P2Y10$^+$ ILC1s, ILC2s and ILC3s in non-inflamed and inflamed intestinal samples of CD patients. White arrows denote indicated ILC subsets expressing P2Y10. Scale bar, 30 μm. Percentages of P2Y10-expressing ILC subsets within each ILC subset are shown in lower panel. n=6 fields for each group. (E) Immunofluorescence staining of intestinal ILCs expressing CD69 or S1PR1 in

non-inflamed and inflamed intestinal samples of CD patients with CD69 or S1PR1 (red), CD127 (green), CD3 (blue) antibodies and DAPI (grey). White arrows indicate intestinal $CD69^+$ and $S1PR1^+$ ILCs. Scale bar, 20 μm. Percentages of $CD69^+$ or $S1PR1^+$ ILCs in $CD127^+$ cells per field are shown in lower panel. n=6 fields for each group. Data are representative of at least three independent experiments and are shown as the means ± SD. Statistical analysis was performed by using unpaired two-tailed Student's t test (*P < 0.05, **P < 0.01, ***P < 0.001, ns, not significant).

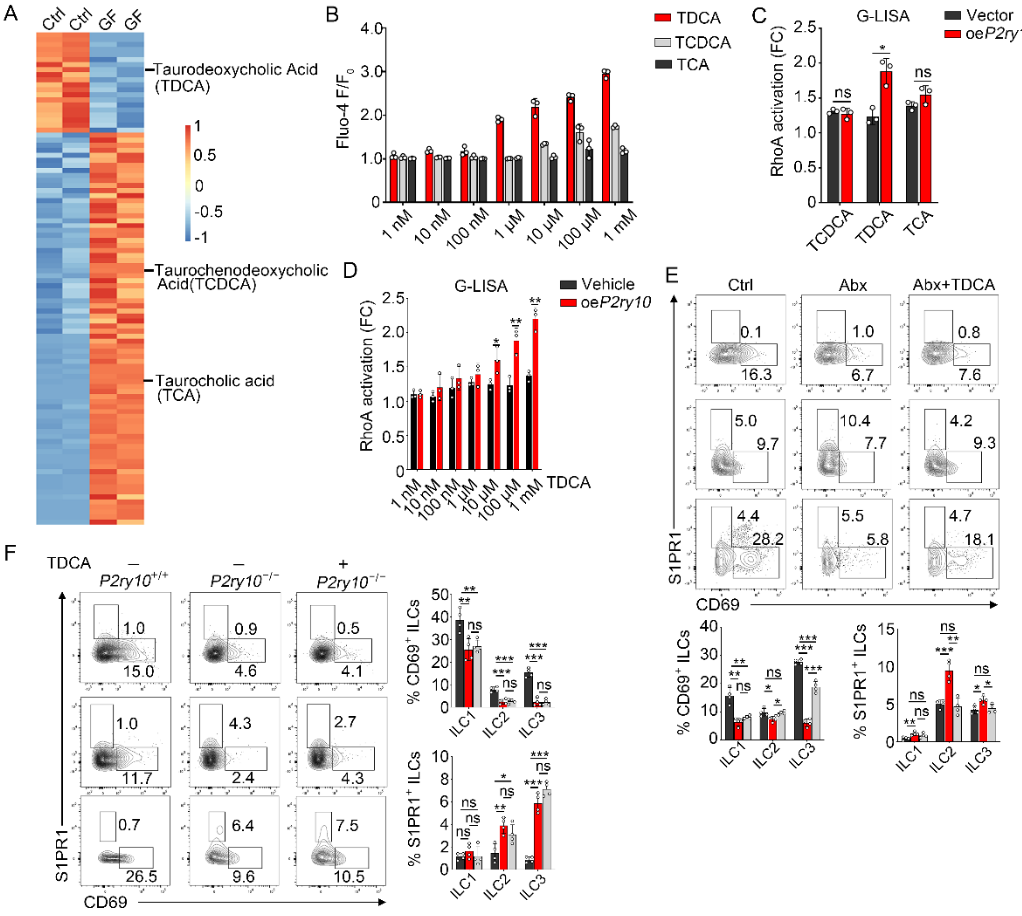

Figure S8. TDCA and P2Y10 engagement activates downstream signaling pathways to mediate tissue residency of ILCs. (A) Metabolome analysis of intestinal contents isolated from Ctrl and GF mice. Heatmap shows significantly differentiated metabolites in the sample group of positive ion patterns. TDCA and its metabolite precursors are marked. (B) *P2ry10* gene was overexpressed in HEK293T. Two days later, calcium indicator fluo-4 AM (2 μM) was loaded and cells were stimulated with different dose of microbiota metabolites. Fluorescent signals of Fluo-4 AM were detected with a Nikon confocal microscopy and subsequently analyzed with NIS Elements AR software. Related fluorescence intensity was calculated as F/F_0 to indicate activation of receptor. The maximum F/F_0 was calculated. n = 4 for each group. (C) *P2ry10* overexpression plasmids and empty vector were transfected into HEK293T cells. Two days later, changes in the levels of active RhoA (GTP-bound) in HEK293T cells stimulated with different microbiota metabolites were detected by the G-LISA kits. (D) Changes in the levels of active RhoA in HEK293T cells stimulated with different doses of TDCA were detected by the G-LISA kits. (E) Mice were treated with TDCA (1 mg/20g body weight, diluted in drinking water) via orally gavage daily for 2 weeks while maintained on Abx drinking. Flow cytometry analysis of S1PR1 and CD69 expression on ILC1s, ILC2s and ILC3s from indicated mice. Frequencies of $S1PR1^{+}$ and $CD69^{+}$ ILC subsets are shown in lower panel. n = 4 for each group. (F) *P2ry10*$^{+/+}$ and *P2ry10*$^{-/-}$ mice kept on normal drinking were orally administrated with PBS or TDCA (1 mg/20g body weight, diluted in drinking water) daily for 2 weeks. Flow cytometry analysis of S1PR1 and CD69 expression on ILC1s, ILC2s and ILC3s from

indicated mice. Frequencies of $S1PR1^+$ and $CD69^+$ ILC subsets are shown in right panel. n = 4 for each group. Data are representative of at least three independent experiments and are shown as the means ± SD. Statistical analysis was performed by using unpaired two-tailed Student's t test (*P < 0.05, **P < 0.01, ***P < 0.001, ns, not significant).

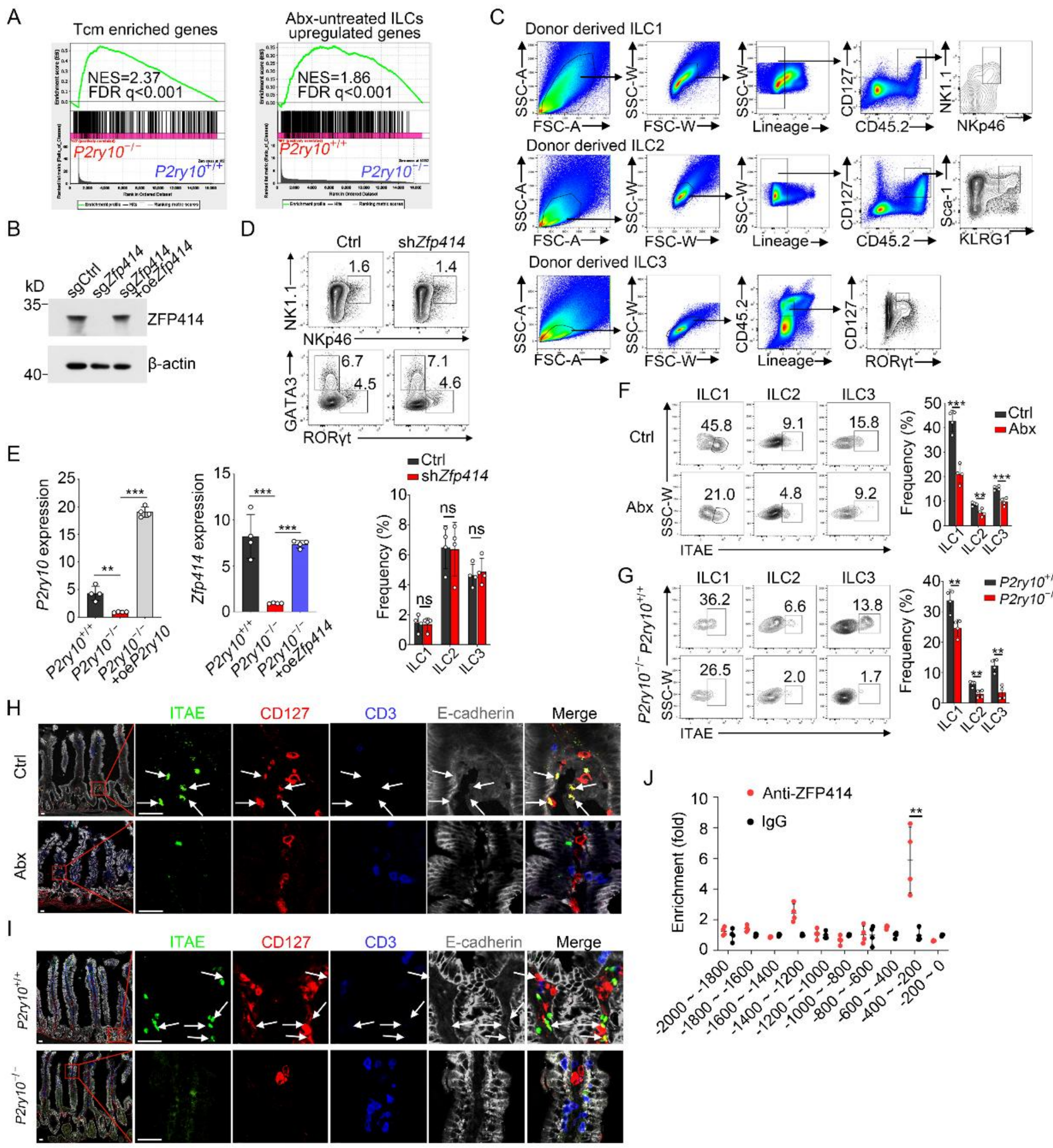

Figure S9. Intestinal ILCs maintain retention via expression of CD69 and ITAE. (A) Gene set enrichment analysis (GSEA) was performed on genes of intestinal ILCs from *P2ry10*$^{+/+}$ and *P2ry10*$^{-/-}$ mice compared with Tcm feature genes or Ctrl ILCs enriched genes dataset. FDR, false discovery rate. NES, normalized enrichment score. (B) Cas9-based *Zfp414* knockout was performed by making chimeras reconstituted with Cas9-expressing BM cells transduced with sg*Zfp414*. Then *Zfp414* was reintroduced into BM cells by lentivirus containing plasmids overexpressing *Zfp414* and BM chimeric mice with rescued *Zfp414* expression were generated. Two months later, donor-derived ILCs (Lin^{-}CD45.2^{+}CD127^{+}) in chimeras were sorted by flow cytometry. The expression of ZFP414 in ILCs was detected by western blot. (C) Gating strategy for donor-derived CD45.2^{+} ILC1s, ILC2s and ILC3s in small intestine from different BM chimeras related to Fig. 5D and 5E. (D) CHILPs (Lin^{-}CD25^{-}CD127^{+}Flt3^{-}α4β7^{+}Id2^{GFP}) were sorted and transduced by lentivirus containing sh*Zfp414*, then cultured under *in vitro* differentiation condition for 14 days. Flow cytometry analysis of indicated ILC subsets was performed

and frequencies were shown in lower panel. n = 4 for each group. (E) CD45.2$^+$ *P2ry10*$^{-/-}$ BM cells were infected with lentivirus containing plasmids overexpressing *P2ry10* or *Zfp414* and 1:1 mixed with CD45.1$^+$ BM cells. The mixed cells were then transplanted into lethally irradiated CD45.1$^+$ recipients. 2 months later, donor-derived ILCs (Lin$^-$CD45.2$^+$CD127$^+$) in chimeras were sorted by flow cytometry. RNA was extracted and overexpression efficiency of *P2ry10* and *Zfp414* was validated by qPCR. Fold changes were normalized to *Actb*. (F, G) Flow cytometry analysis of ITAE expression on ILC1, ILC2 and ILC3 in intestines from Abx-treated (F), *P2ry10*$^{-/-}$ (G) and their corresponding control mice. Numbers in flow cytometry plots represent percentages of indicated ILC subsets. Frequencies and numbers of indicated ILC subsets are shown in right panel. n = 4 for each group. (H, I) Immunofluorescence staining of the interaction of integrin αE-expressing intestinal ILCs with E-cadherin$^+$ epithelial cells in Abx-treated (H), *P2ry10*$^{-/-}$ mice (I) and their control littermates. ITAE (green), CD127 (red), CD3 (blue) and E-cadherin (grey) antibodies were used. White arrows indicate ITAE$^+$ ILCs adhering to intestinal epithelia. Scale bar, 20 μm. (J) Enrichment of ZFP414 on *Itage* gene promoter was analyzed by ChIP assay with anti-ZFP414 antibody. n = 4 for each group. Data are representative of at least three independent experiments and are shown as the means ± SD. Statistical analysis was performed by using unpaired two-tailed Student's t test (*P < 0.05, **P < 0.01, ***P < 0.001, ns, not significant).

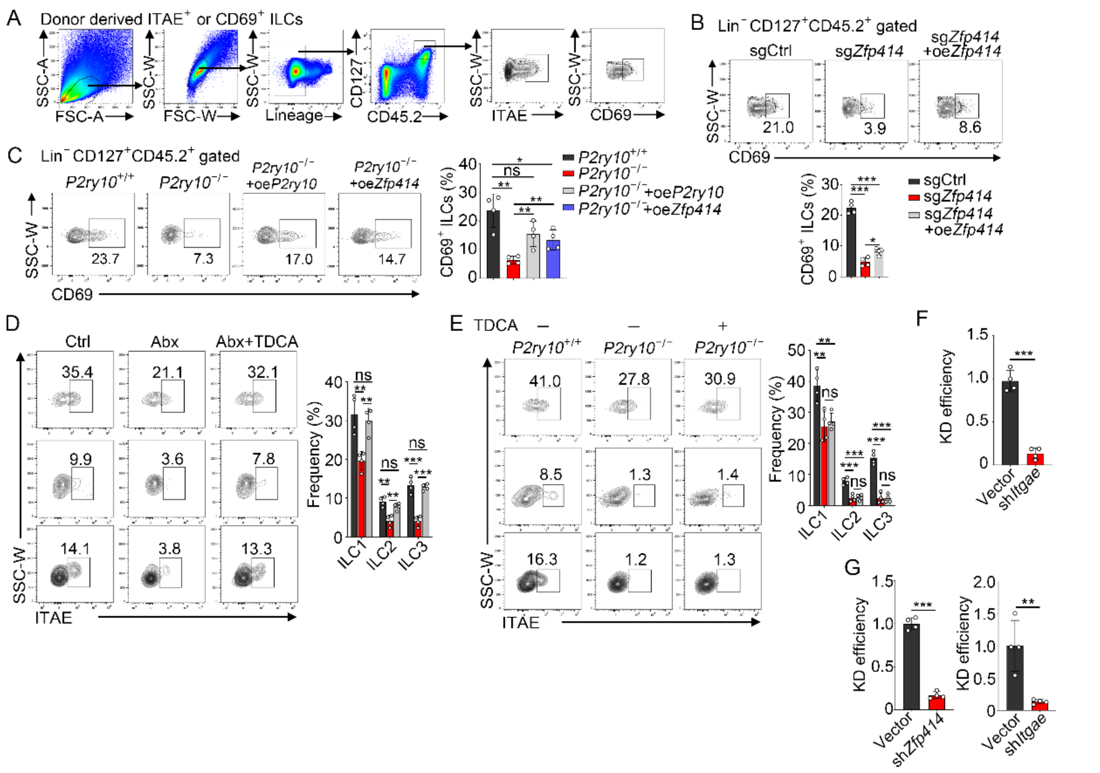

Figure S10. TDCA-P2Y10-ZFP414 regulates expression of CD69 and ITAE on intestinal ILCs. (A) Gating strategy for donor-derived CD45.2$^+$ ILCs expressing ITAE and CD69 in small intestine from different BM chimeras related to Fig. 6E, 6F, S10B and S10C. (B) BM chimeric mice with *Zfp414* knockout and reintroduction were constructed as in Figure S9B. Flow cytometry analysis of CD45.2$^+$ intestinal ILCs expressing CD69 in different BM chimeras. Numbers in flow cytometry plots represent percentages of indicated ILCs in each gate. Frequencies of intestinal CD69$^+$ ILCs in indicated BM chimeras are shown in lower panel. n = 4 for each group. (C) BM chimeric mice were generated as in Figure S9E. Flow cytometry analysis of CD45.2$^+$ intestinal ILCs expressing CD69 in different BM chimeras. Numbers in flow cytometry plots represent percentages of indicated ILCs in each gate. Frequencies of intestinal CD69$^+$ ILCs in indicated BM chimeras are shown in right panel. n = 4 for each group. (D) Mice were treated with TDCA (1 mg/20g body weight, diluted in drinking water) via orally gavage daily for 2 weeks while maintained on Abx drinking. Flow cytometry analysis of ITAE expression on ILC1s, ILC2s and ILC3s from indicated mice. Frequencies of ITAE$^+$ ILC subsets are shown in right panel. n = 4 for each group. (E) *P2ry10*$^{+/+}$ and *P2ry10*$^{-/-}$ mice kept on normal drinking were orally administrated with PBS or TDCA (1 mg/20g body weight, diluted in drinking water) daily for 2 weeks. Flow cytometry analysis of ITAE expression on ILC1s, ILC2s and ILC3s from indicated mice. Frequencies of ITAE$^+$ ILC subsets are shown in right panel. n = 4 for each group. (F) ILC progenitors were isolated and transduced with lentivirus containing sh*Itgae*. Subsequently, in vitro differentiation experiments were conducted. Differentiated ILCs were collected and RNA was extracted. Knockdown efficiency of *Itgae* was validated by qPCR. (G) Intestinal lymphocytes were isolated from *P2ry10*$^{+/+}$, *P2ry10*$^{+/+}$; sh*Zfp414* or *P2ry10*$^{+/+}$; sh*Itgae* mice, then RNA was extracted. Knockdown efficiency of *Zfp414* and *Itgae* was validated by qPCR. Data are

representative of at least three independent experiments and are shown as the means ± SD. Statistical analysis was performed by using unpaired two-tailed Student's t test (*P < 0.1, **P < 0.01, ***P < 0.001, ns, not significant).

Table S1. sgRNA sequences used in this study

Target gene	sgRNAs
P2ry10 (Forward)	5’- GGTCCTGTGTCCAAAGTGTA -3’
P2ry10 (Reverse 1)	5’- TTTCCAGGTACAACATGCAA -3’
P2ry10 (Reverse 2)	5’- ACAGAGACTTGCAAGGCATA -3’
Zfp414 (Forward)	5’- TGCACCAGGAGGAACCATCC -3’
Zfp414 (Reverse)	5’- TGTGCTAGATCACGAACACT -3’

sgRNAs were designed according to online available tool (https://www.zlab.bio/resources), and purchased from Sangon Company.

Table S2. Primer Sequences used for qPCR

Gene	Sequences
P2ry10 (Forward)	5’-TGCTGAACTTGCTGGATTTGTG-3’
P2ry10 (Reverse)	5’-CACACATGAAAACCATCCGCA-3’
Gpr132 (Forward)	5’-GTGCCATTGTGGATCATCTACA-3’
Gpr132 (Reverse)	5’-CTCTCCAGTGCATAGACCACG-3’
Adgre5 (Forward)	5’-GAACTGTGCCAAGTGGTGC-3’
Adgre5 (Reverse)	5’-TGGCAAAGTCTCCACAGGAA-3’
Ccr5 (Forward)	5’-TTTTCAAGGGTCAGTTCCGAC-3’
Ccr5 (Reverse)	5’-GGAAGACCATCATGTTACCCAC-3’
Cxcr6 (Forward)	5’-GAGTCAGCTCTGTACGATGGG-3’
Cxcr6 (Reverse)	5’-TCCTTGAACTTTAGGAAGCGTTT-3’
Ccrl2 (Forward)	5’-GCCCCGGACGATGAATATGAT-3’
Ccrl2 (Reverse)	5’-CACCAAGATAAACACCGCCAG-3’
Cxcr4 (Forward)	5’-GACTGGCATAGTCGGCAATG-3’
Cxcr4 (Reverse)	5’-AGAAGGGGAGTGTGATGACAAA-3’
F2r (Forward)	5’-TGAACCCCCGCTCATTCTTTC-3’
F2r (Reverse)	5’-CCAGCAGGACGCTTTCATTTTT-3’
Gpr171 (Forward)	5’-TCAAATGTAGGTTGCATGGAGTT-3’
Gpr171 (Reverse)	5’-GACAGCGTGGTAGGGAACAAA-3’
Gpr65 (Forward)	5’-ATGGCGATGAACAGCATGTG-3’
Gpr65 (Reverse)	5’-ACGCATAAAGATCCGATGTTGG-3’
Cd69 (Forward)	5’-TTCACATCTGGAGAGAGGGCA-3’
Cd69 (Reverse)	5’-AACACAGCCCAAGGGATAGA-3’
Sell (Forward)	5’-TACATTGCCCAAAAGCCCTTAT-3’
Sell (Reverse)	5’- CATCGTTCCATTTCCCAGAGTC-3’
Itgae (Forward)	5’-GTATGACAGTCCTTCCCAGCA-3’
Itgae (Reverse)	5’-TACGGTCAGGTCAACCACAG-3’
Itga1 (Forward)	5’-CCTTCCCTCGGATGTGAGTCA-3’

Itga1 (Reverse)	5’-AAGTTCTCCCCGTATGGTAAGA-3’
Itgb7 (Forward)	5’-ACCTGAGCTACTCAATGAAGGA-3’
Itgb7 (Reverse)	5’-CACCGTTTTGTCCACGAAGG-3’
Ccr9 (Forward)	5’-CTTCAGCTATGACTCCACTGC-3’
Ccr9 (Reverse)	5’-CAAGGTGCCCACAATGAACA-3’
Ccr7 (Forward)	5’-TGTACGAGTCGGTGTGCTTC-3’
Ccr7 (Reverse)	5’-GGTAGGTATCCGTCATGGTCTTG-3’
Ly6c (Forward)	5’-GCAGTGCTACGAGTGCTATGG-3’
Ly6c (Reverse)	5’-ACTGACGGGTCTTTAGTTTCCTT-3’
Tcf7 (Forward)	5’-GTACATGGAGAAGCCGAGGG-3’
Tcf7 (Reverse)	5’-GGGTAGGGCATGAGCAGATT-3’
Klf2 (Forward)	5’-CTCAGCGAGCCTATCTTGCC-3’
Klf2 (Reverse)	5’-CACGTTGTTTAGGTCCTCATCC-3’
Ccr6 (Forward)	5’-CCTGGGCAACATTATGGTGGT-3’
Ccr6 (Reverse)	5’-CAGAACGGTAGGGTGAGGACA-3’
Zfp414 (Forward)	5’-CCTTTTACCTCTGCCCCCAC-3’
Zfp414 (Reverse)	5’-GGTAGCGCCTTGACTCTTCT-3’
Batf (Forward)	5’-CTGGCAAACAGGACTCATCTG-3’
Batf (Reverse)	5’-GGGTGTCGGCTTTCTGTGTC-3’
Thap7 (Forward)	5’-TCCCAAGAAAGACAACCCCAG-3’
Thap7 (Reverse)	5’-TTCCTCGAAGTGTTTGGAGCA-3’
Junb (Forward)	5’-TCACGACGACTCTTACGCAG-3’
Junb (Reverse)	5’-CCTTGAGACCCCGATAGGGA-3’
Zfp688 (Forward)	5’-CTCCGAGAGGAGACATTCCAA-3’
Zfp688 (Reverse)	5’-CTGGCCTGAATATCGGTGTTT-3’
Trp53 (Forward)	5’-GCGTAAACGCTTCGAGATGTT-3’
Trp53 (Reverse)	5’-TTTTTATGGCGGGAAGTAGACTG-3’
Zfp707 (Forward)	5’-GGGCCTTATTACCCGTCTGG-3’
Zfp707 (Reverse)	5’-CGGCTCCTATCCGTGAATGG-3’

Maf1 (Forward)	5’-CTTTGAGGCCATCAACTCACA-3’
Maf1 (Reverse)	5’-CTGCACTTGTCGCTCAGAG-3’
Itgb3bp (Forward)	5’-GAGCCCATTTTCTTCTCCCG-3’
Itgb3bp (Reverse)	5’-GCAACACCATGAATCCATCCC-3’
Itgb2 (Forward)	5’-CAGGAATGCACCAAGTACAAAGT-3’
Itgb2 (Reverse)	5’-CCTGGTCCAGTGAAGTTCAGC-3’
Itgb1bp1 (Forward)	5’-GAGGCGAGTCCTGGAAACAA-3’
Itgb1bp1 (Reverse)	5’-GCTGCTGCTGTGTCTCTTCT-3’
P2RY10 (Forward)	5’-GCGTTGGTCGGGATGATTAC-3’
P2RY10 (Reverse)	5’-ACACATGAACACCATCCGCA-3’
CD69 (Forward)	5’-CTTTGCATCCGGAGAGTGGA-3’
CD69 (Reverse)	5’-CAGCACACAGGACAGGAACT-3’
S1PR1 (Forward)	5’-TTCCACCGACCCATGTACTAT-3’
S1PR1 (Reverse)	5’-GCGAGGAGACTGAACACGG-3’
ITAE (Forward)	5’-CCTCAAGAGGTCATCTGCTCA-3’
ITAE (Reverse)	5’-GGTGTGGGTCTCGTTGGC-3’
Actb (Forward)	5’-GGCTGTATTCCCCTCCATCG-3’
Actb (Reverse)	5’-CCAGTTGGTAACAATGCCATGT-3’
ACTB (Forward)	5’-CATGTACGTTGCTATCCAGGC-3’
ACTB (Reverse)	5’-CTCCTTAATGTCACGCACGAT-3’

Table S3. Primers for ChIP assay in this study

Gene	Sequences
Itgae pro#1 (Forward)	5'- CCCTAATCTAGGTGCCTGAAGCTCT -3'
Itgae pro#1 (Reverse)	5'- GCACAGGGCACAGGTTACTTT -3'
Itgae pro#2 (Forward)	5'- GTCTTGTTGCTTGCGGAGTT -3'
Itgae pro#2 (Reverse)	5'- TGATCCAAGGACATTTGGTAG -3'
Itgae pro#3 (Forward)	5'- GTGCCACTGCAGCTTGTGAC -3'
Itgae pro#3 (Reverse)	5'- ACAGTTCACCACCACCACCC -3'
Itgae pro#4 (Forward)	5'- CCAAAGCTAACAGCGTGGAA -3'
Itgae pro#4 (Reverse)	5'- TGTAAGCGAACATTCTGGAGTA -3'
Itgae pro#5 (Forward)	5'- GAGGTTTGATGCCCAGCTTT -3'
Itgae pro#5 (Reverse)	5'- AAGATGGGCTGCTTTGAACC -3'
Itgae pro#6 (Forward)	5'- CTTGAACTCAGTCTTCCCACC -3'
Itgae pro#6 (Reverse)	5'- TACCAACAGGGGCAGTCATA -3'
Itgae pro#7 (Forward)	5'- GCCATGAGGTCTACCTCTTCT -3'
Itgae pro#7 (Reverse)	5'- TGTCTGCTCCTGTACTAAACAC -3'
Itgae pro#8 (Forward)	5'- CTGTGAGGCCACTTTCTATTT -3'
Itgae pro#8 (Reverse)	5'- GTGGTCGTTCTGAGTGTCTTT -3'
Itgae pro#9 (Forward)	5'- GGTGAGTCTAGTGAGTGATGGAA -3'
Itgae pro#9 (Reverse)	5'- CAGAGTGCTGGGAATAAAGGT -3'
Itgae pro#10 (Forward)	5'- CAGCCTGGTCTACAGAGTGAG-3'
Itgae pro#10 (Reverse)	5'- GAAAGTACTCAGCTTCCCTTC -3'
